# Restorative effects of *Momordica charantia* extract on cerebellar GFAP and NGF expression in pregnant diabetic rats and their offspring

**DOI:** 10.1371/journal.pone.0321022

**Published:** 2025-04-04

**Authors:** Amoura M. Abou-El-Naga, Hend Abd EL-Halim Mansour, Mamdouh R. El-Sawi, Mai Alaa El-Dein, Yasmin M. Tag, Reham A. Ghanem, Manar A. Shawki

**Affiliations:** 1 Zoology Department, Faculty of Science, Mansoura University, Mansoura, Egypt; 2 Zoology and Entomology Department, Faculty of Science, Al-Azhar University, Cairo, Egypt; 3 Oral BiologyDepartment, Faculty of Oral and Dental Medicine, Delta University for Science and Technology, Gamsa, Egypt; Helwan University, EGYPT

## Abstract

Maternal diabetes mellitus is linked to neurobiological and cognitive impairments, increasing the risk of brain and cerebellar defects in diabetic pregnant rats and their offspring. *Momordica charantia* (bitter melon) possesses antidiabetic properties due to its bioactive compounds, including phenolics, alkaloids, proteins, steroids, inorganic compounds, and lipids. Forty pregnant rats were randomly assigned to four groups: control; *M charantia* (BM); diabetic (DM); and diabetic treated with *M charantia* (BM+DM). Diabetic maternal rats showed significantly elevated serum glucose, insulin, leptin, and homeostasis model assessment of insulin resistance (HOMA-IR) levels, with a concomitant decrease in insulin sensitivity check index (QUICKI), glucose transporter 4 (GLUT4), adenosine monophosphate-activated protein kinase (AMPK), acetylcholine (ACh), and dopamine. Oxidative stress markers in cerebellar tissue indicated increased malondialdehyde (MDA) and decreased glutathione (GSH) levels. Cerebellar tissue analysis revealed significantly reduced superoxide dismutase (SOD), catalase (CAT), B-cell lymphoma 2 (Bcl-2), and nerve growth factor (NGF), while Bcl-2-associated X protein (BAX) and glial fibrillary acidic protein (GFAP) were elevated. Histological and ultrastructural analysis of the diabetic maternal cerebellum showed moderate vacuolation of the neuropil in all cerebellar cortical layers, along with Purkinje cell degeneration and necrosis, including Nissl substance loss. Offspring of diabetic mothers exhibited multifocal Purkinje cell loss, empty baskets, and cerebellar cortical dysplasia with abnormal tissue development and organization. In conclusion, *M. charantia* supports central nervous system health in diabetic pregnant rats and their offspring by enhancing antioxidant markers, regulating GFAP and NGF, and mitigating apoptosis, ultimately improving cerebellar pathology and neural development.

## 1. Introduction

Maternal glucose is the principal macronutrient that sustains fetal growth and health. Prolonged exposure of the fetus to hyperglycemia from the early stages of pregnancy accelerates the maturation of the stimulus–secretion coupling mechanism in β cell autoimmunity, which leads to early hyperinsulinemia [1]. This could be due to reduce insulin utilization or secretion or both [2]. Diabetes can cause devastating macrovascular complications as well as microvascular complications such as diabetic neuropathy. These contribute to increased mortality and poor quality of life [3]. Type 1 diabetes mellitus (T1DM), type 2 diabetes mellitus (T2DM), and gestational diabetes mellitus (GDM) are the three main types of diabetes [4]. Gestational diabetes increases the risk of abortion, foetal death, and new-born mental malformations [5]. **Hernández-Fonseca et al.** [6] has demonstrated that brain structure and cognitive function have been changed correlated to DM in adult rats with STZ-induced diabetes, in addition to an increase in the apoptosis of pyramidal neurons in the brain and Purkinje cells in the cerebellum. Moreover, it was proven that DM during pregnancy adversely affects the hippocampal neuronal density, as well as cortical and cerebellar white matter thickness, in neonatal rats [7].

The late embryonic stage marks the start of cerebellar development, which continues to change structurally long after birth. When exposed to diabetes, the cerebellum is especially vulnerable to developmental defects, which can result in oxidative stress. An imbalance between the production of reactive oxygen species and antioxidant defenses causes oxidative stress, which can interfere with signaling pathways and result in abnormalities and malfunction of the cerebellum. Some plants like *M charantia* contain antioxidants that might help reduce toxicity in the developing cerebellum [8].

In Asia and Africa, *Mcharantia* (bitter melon, BM) is a widely distributed vegetable and belongs to the family Cucurbitaceae. It goes by the name balsam pear and karela as well [4]. Although bitter melon is scorned for its bitter taste, the fruit has plenty of vital nutrients. Several phytomedicines obtained from *M charantia* have efficacy against diabetes [9]. Bitter melon is commonly has medicinal nutrients including phenolic acids, flavonoids, triterpenoids, sterols, triterpene, glycoside, lectins, and proteins that show high potential antioxidant and anticancer activities with little negative impact [10]. Bitter melon extract didn’t protect against β-cell dysfunction; rather, it has shown hypoglycemic effects and can enhance insulin sensitivity in rats with T2DM [11].

This article aimed to investigate the therapeutic effectiveness of *M charantia* as an anti-diabetic plant. Additionally, it examined the antioxidant and anti-inflammatory properties that influence the regulation of GFAP and NGF expression, impacting central nervous system health and neural development in diabetic pregnant rats and their offspring.

## 2. Methods

### 2.1. Chemicals

Streptozotocin (STZ) and nicotinamide (NA) were acquired from Sigma‒Aldrich Company for Chemicals (St. Louis, Missouri, USA). Analytical grade reagents and chemicals were utilised in all other instances.

### 2.2. Animals and ethics approval

The Egyptian Institute for Serological and Vaccine Production, located in Helwan, Egypt, provided forty adult female and twenty adult three-month-old male Sprague Dawley rats (body weight: 150 ±  20 g). The female rats were randomly and equally distributed into two main groups: the nondiabetic and diabetic pregnant rats, each of them further divided into two groups 6 rats/group. The animals were kept in the animal facility. The bedding in the stainless-steel cages housing the rats was changed daily, and the cages consisted of wood chips. The rats were housed in a climate-controlled space with a 12-hour light/dark cycle. Two weeks before the trials started, all the rats were allowed to acclimate to the location. Throughout the trial, all the rats were fed a regular diet and had unlimited access to water. This study was approved by the Ethics Committee of the Laboratory Animals of the Faculty of Science, Mansoura University (serial No. Sci-Z-M-2020-80). The experiment adhered to the National Institutes of Health’s guidelines outlined in their publication (NIH Publication No. 85-23, revised 1996) regarding the care and utilization of laboratory animals.

### 2.3. Diabetes induction

Twenty minutes prior to the intraperitoneal (i.p.) injection of STZ (55 mg/kg), female rats were given an i.p. injection of NA (100 mg/kg) to induce experimental diabetes [12]. Fresh preparations of NA and STZ were made in citrate buffer (pH 4.5) [13]. Seventy-two hours later, hyperglycemia was confirmed. Accu-Chek Blood Glucose Metres (Roche Diagnostics, Mannheim, Germany) were used to measure the animal’s blood glucose level from a blood drop taken from its tail. For the purposes of the studies, diabetic rats were defined as those whose blood glucose levels at fasting were more than 250 mg/dl. Some female rats could not tolerate the injection of the Streptozotocin, leading to their death.

### 2.4. Estrus cycle and mating

The female had to go through at least two consecutive estrous cycles before using the vaginal smear technique to determine her oestrus cycle stage. The study excluded females who failed to mate within one oestrus cycle. Diabetic female rats were bred with nondiabetic healthy males (1:1). All females were checked for vaginal plugs [14]. On gestational day 0, the vaginal plug was visible on the 1st day. Additionally, due to the diabetic condition, some other females did not conceive despite undergoing the mating process multiple times. As a result, these animals were excluded from the experiment. Due to the reasons mentioned the number of rats was reduced to 24 out of a total of 40 included at the beginning of the experiment.

### 2.5. *Momordica charantia* preparation

*M. charantia* fruit powder was purchased from Bixa Botanical International, NineLife, UAE. *M. charantia* powder was made, with a daily dosage of 250 mg/kg b.wt. The dose was dissolved in warm distilled water before administration [4,15]. An oral stomach tube was used to administer a freshly prepared aqueous solution containing 37.5 mg/ml of dist. water at a dose of 250 mg/kg of body weight.

### 2.6. Experimental design

The nondiabetic pregnant rats were subdivided into the **control group (C)** without any treatment, and the **BM** group included pregnant rats treated orally via gastric tubes at a dose of 250 mg/kg b.wt. of *M. charantia* fruit powder dissolved in warm distilled water daily until birth for 22 days. Pregnant diabetic rats were subdivided into **DM** group: pregnant rats were intraperitoneally injected with 100 mg/kg NA and then i.p. injected with 55 mg/kg STZ for 20 min before mating. **BM+DM** group: pregnant diabetic rats were orally supplemented with *M. charantia* fruit powder at a dose of 250 mg/kg body weight dissolved in warm distilled water daily until birth for 22 days. The schedule was repeated from the 1^st^ day of pregnancy beginning (the day of pregnancy confirmation) until the 21^st^ gestational day. The day after birth, all the mother rats in all groups were weighed at the end of the experiment, fasted for an entire night, and given an intraperitoneal injection of 6 mg/kg xylazine and 75 mg/kg ketamine [16]. Then all animals were sacrificed by cervical dislocation(quick death). Efforts were taken to minimize animals suffering. This included careful handling of animals, provision of appropriate environmental enrichment in their housing, and regular monitoring for signs of pain or distress. Interventions were applied immediately when needed to alleviate discomfort. This method was chosen to ensure a quick and painless procedure.

### 2.7. Offspring defects in the control and the other experimental groups

At the end of the study, the number of pregnant rats in each group was calculated. Female rats that did not conceive over three consecutive estrous cycles were excluded. Immediately after birth, offspring’s measurements were taken which, included the number of a live newborns, their weights in grams, and their lengths in centimeters. Additionally, the number of corpora lutea in the ovaries was also calculated.

### 2.8. Blood collection

For biochemical analysis, blood samples were obtained, coagulated at room temperature, and centrifuged for 10 minutes at 3000 rpm to produce clear, aliquots were made in many Eppendorf tubes and stored at - 20 °C for subsequent biochemical analyses in serum.

### 2.9. Biomarkers analyses

Serum glucose level was estimated according to the technique used by **Trinder** [17], using kit from Biodiagnostic Co. Dokki, Giza, Egypt. The homeostasis model assessment of IR (HOMA-IR) and insulin sensitivity check index (QUICKI) were calculated by equations described by **Katz et al.** [18], respectively. The level of GLUT4 in serum was determined quantitatively using a rat ELISA kit provided by CUSABIO (Fannin, Houston, USA), Catalog Number. CSB-E13908r. The level of AMPK in serum was measured using kit from RnDSystems (USA & Canada), Catalog Number: DYC3197-2(2 plates) DYC3197-5 (5 plates). The level of insulin in serum was determined using RAT insulin ELISA Kit provided by BioVendor R&D (Asheville, North Carolina, USA), Catalogue number: RTC018R. The level of leptin in serum was estimated by ELISA using rat leptin kit procured from MyBioSource (San Diego, California, USA). Leptin levels were estimated according to the manufacturer’s instructions and expressed as ng/ml protein.

The level of acetylcholine in cerebellum was determined using Rat AChE ELISA Kit provided by MyBioSource (San Diego, California, USA), Catalog No: MBS2501434. The level of dopamine in serum was determined using Mouse/ Rat Dopamine ELISA Assay Kit provided by Eagle Biosciences (20A Northwest Blvd., Suite 112, Nashua, NH 03063, USA), Catalog Number: DOU39-K01 (1 x 96 wells). Malondialdehyde (MDA) concentration in cerebrum of control and different maternal groups was determined using Biodiagnostic Kit, Egypt [19]. The content of GSH in cerebrum of control and different maternal groups were estimated according to the colorimetric method of **Beutler et al.** [20], using kit from Biodiagnostic Co. Dokki, Giza, Egypt. Activity of SOD in cerebrum of control and different maternal groups were assayed by the procedure of **Nishikimi et al.** [21], using kit from Biodiagnostic Co. Dokki, Giza, Egypt. Activity of CAT in cerebellum homogenate were determined by the colorimetric method of **Aebi** [22], using kit from Biodiagnostic Co. Dokki, Giza, Egypt. B-cell lymphoma-2 associated (BAX) level in the rat cerebrum was estimated quantitatively using FITC Anti-Bax antibody [T22-A] ab139543 obtained from Abcam (Waltham, Boston) and B- cell lymphoma 2 (Bcl2) level in the rat cerebrum was estimated quantitatively using Bcl-2 Monoclonal Antibody (10C4), FITC, eBioscience™ obtained from Invitrogen (Waltham, Massachusetts, USA), Catalog # 11-6992-42.

### 2.10. Isolation of cerebellum and cerebellum homogenate preparation

Mother rats and their offspring’s were dissected promptly, then the cerebellum of all of them was excised immediately, washed with cold saline (0.9%), cleaned, and dried with lint free tissue. A suitable weight (0.3 g) of left cerebellum was homogenized. The homogenate was then followed by centrifugation at 12,000 xg for 20 min at 4 °C. The supernatant for each cerebellum was collected to be used for the subsequent biochemical parameters [23].

### 2.11. Histopathological studies and microscopic investigation

The maternal and neonatal cerebellar tissue samples were carefully fixed in neutral buffered formalin solution (10%). Dehydration process was carried out in an ascending grades of ethyl alcohol, then cleared in xylene and embedded in a highly purified paraffin wax. 5–7 µm sections were made, hydrated in descending series of ethanol and then stained with hematoxylin and eosin stain. The stained sections then were examined and photographed using Olympus light microscope with a camera (Amscope MU1000) to detect histopathological alterations, Purkinje cell count was performed using 5different fields/sample, then statistical analysis was performed [24].

### 2.12. Immuno-histochemical (IHC) investigations

Paraffin-embedded cerebellar sections of mothers and their offspring’s were deparaffinized in xylene and then processed for IHC staining using the labeled streptavidin–biotin immunoperoxidase technique according to a previously published guideline [25]. Sections were then submerged in 10 mM citrate buffer (pH 6), heated to boiling in a Gibson oven (USA) on full power, and then allowed to cool for a minimum of 10–20 minutes at room temperature (a microwave approach was used to increase the exposure of the antigen). Endogenous peroxidase activity was inhibited by using 3% hydrogen peroxide H2O2 in phosphate buffer saline (PBS, 10 mM sodium phosphate, 140 mM sodium chloride, pH 7.2). After washing the slides in phosphate buffer saline three times for two minutes each, the immunohistochemistry procedure was initiated. Blocking serum was incubated on sections for ten minutes in order to prevent unspecific binding that could arise from an electrostatic or hydrophobic contact between the antibody and tissue component. The blocking serum that was in excess was eliminated. Primary antibodies against GFAP and NGF (BioGenex, catalogue: AN783-5M and AN738-5M, respectively) were diluted 1:100 and incubated on slides for an entire night at 4°C. After a PBS wash, slides were incubated for one hour at a ratio of 1:200 with biotinylated secondary antibody, followed by a PBS wash at a ratio of 1:1000 with streptavidin-HRP. Following that, Mayer’s Hematoxylin (Sigma Aldrich, USA) was utilized as a counterstain for the sections for 1 minute at ambient temperature, after which the slides were analyzed using optical microscopy. Protein expression was identified in the cerebellar tissues. A brown color signifies positive messages. The evaluation of GFAP and NGF labelling indices relied on staining intensity and the ratio of positive cells, with five fields per specimen selected at random. Results were expressed as the average count of positive cells per specified region, using ImageJ Software 1.42q on a 64-bit Windows 10 installation.

### 2.13. Ultrastructural examination

Mothers’ and neonatal cerebellar fragments were immediately isolated and preserved in 4% glutaraldehyde in Dulbecco’s modified phosphate-buffered saline. Following a one-hour soak at room temperature in 1% osmium tetroxide, the samples were cleaned and next fixed. Following ethanol gradient dehydration, propylene oxide treatment, and Epon resin (Epson 812; FlukaChemie, Switzerland) embedding, the fixed samples were ultrathinly sectioned (60–70 nm) for Transmission Microscopy analysis. The Faculty of Agriculture, Mansoura University, Egypt used a JEOL 2100 TEM to study ultrathin sections that had been cut with a diamond knife on a LKB microtome, mounted on grids, dyed with uranyl acetate and lead citrate, and observed at 80 KV.

### 2.14. Statistical analyses

Graph Pad Prism 7.0 (GraphPad Software Inc., San Diego, California, USA) was used for all the statistical analyses. The findings are shown as the mean ±  the standard error of the mean (SEM) for the six subjects. One-way analysis of variance (ANOVA) was used for statistical comparisons, and the Neuman-Keuls post hoc test was subsequently used [26]. When the P value was ≤  0.05, a significant difference was considered.

## 3. Result

### 3.1. Offspring defects in the control and the other experimental groups

**Fig 1:**
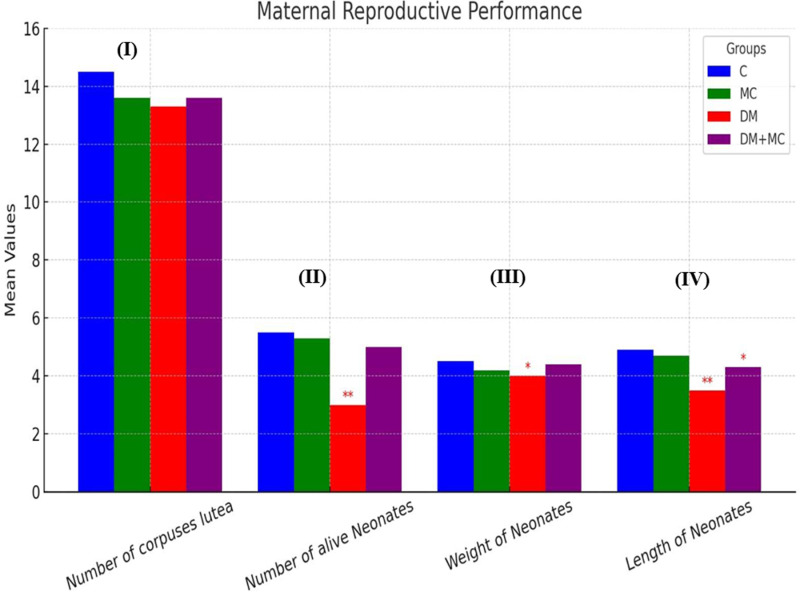
Effect of DM, and/or *M. charantia* on the (I) number of corpuses lutea; (II) number of neonates, (III) weight of neonates; (IV) length of neonates levels in the control and different maternal groups. Values are expressed as means ±  SEM; (n =  6); (Significant, ^* ^*P* < 0.05), (High significant, ^**^P <  0.01).

In the current study there was no significant difference in the number of corpuses lutea in all treated groups. The diabetic group exhibited present of decrease in the number of corpora lutea (−8.28%), while the MC and DM+MC groups showed similar changes, both with a − 6.2% reduction compared to the control group. The DM group had a significantly lower number of a live newborns compared to the other groups. However, treatment with bitter melon extract led to an improvement in the number of a live offspring. The DM group experienced the largest reduction in the number of live newborns (−45.45%), while MC group showed a minimal decrease (−3.64%), and the diabetic group treated with bitter melon extract exhibited a moderate present of reduction (−9.09%) compared to the control group. The DM group also showed a significant reduction in neonatal weight, whereas the MC and DM+MC groups demonstrated an increase in neonatal weight compared to the diabetic group. The diabetic group had an − 11.1% present of decrease in neonatal weight, while the DM+MC group showed a − 6.67% decrease compared to the control group. The MC group had the smallest reduction, at − 2.22%. Significant reductions in neonatal length were observed in the diabetic group, while the MC and DM+MC groups showed improvements in neonatal length compared to the diabetic group. The DM group experienced a − 28.57% decrease in neonatal length, while the DM+MC group showed a − 12.2% decreases and MC group had the smallest reduction in neonatal length, at − 4.08% compared to the control group as shown in Fig 1.

### 3.2. Serum glucose, carbohydrate metabolism, and hormone analyses

**Fig 2:**
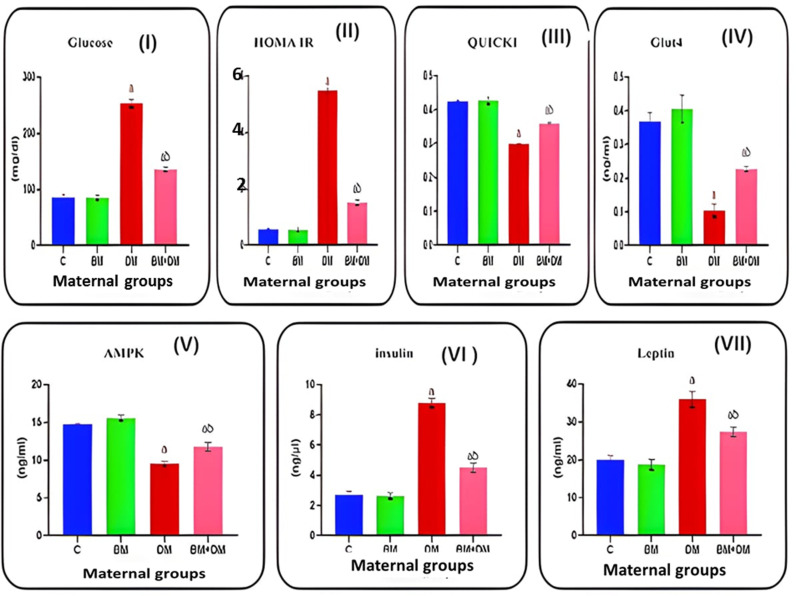
Effect of DM, and/or *M. charantia* on the (I) glucose; (II) HOMA IR, (III) QUICKI levels; (IV) GLUT4; (V) AMPK; (VI) insulin; (VII) leptin levels in the control and different maternal group in the different groups. Values are expressed as means ±  SEM; (n =  6); (a: Significant as compared with the control, *P* < 0.05), (ab: Significant as compared with DM group, *P* <  0.05).

The induction of diabetes led to a significant increase in the levels of serum glucose (253^a^ ± 7.1), HOMA-IR (5.5^a^ ± 0.069), insulin (8.8^a^ ± 0.3), and leptin (36^a^ ± 2.10) compared to the control group. While diabetic pregnant rats treated with *M. charantia* showed a significant reduction in serum glucose (135^ab^ ± 3.8), HOMA-IR (1.5^ab^ ± 0.1), insulin (4.5^ab^ ± 0.31), and leptin (27^ab^ ± 1.20) levels compared to untreated diabetic rats. In contrast, untreated diabetic rats displayed significantly lower serum levels of QUICKI (0.3^a^ ± 0.001), GLUT4 (0.1^a^ ± 0.019), and AMPK (9.5^a^ ± 0.32), compared to control pregnant rats. However, daily oral administration of *M. charantia* (250 mg/kg) until birth significantly improved QUICKI (0.36^ab^ ± 0.004), GLUT4 (0.23^ab^ ± 0.007), and AMPK (12^ab^ ± 0.56) levels compared to untreated diabetic mothers as shown in Fig 2.

### 3.3. Neurotransmitters, oxidative stress, and apoptotic markers

Diabetic mothers exhibited significantly reduced cerebellar acetylcholine (1.2^a^ ±  0.044) and dopamine (0.57^a^ ±  0.092) levels in serum compared to control mothers. Oral treatment of diabetic rats with *M. charantia* resulted in a significant increase in Ach (2.3^ab^ ±  0.035) and dopamine (1.1^ab^ ± 0 .087) levels compared to untreated diabetic rats.

Moreover, diabetic pregnant rats had significantly elevated malondialdehyde concentrations (1096^a^ ±  37) in cerebellar homogenates, indicating increased oxidative stress, compared to control mothers. Additionally, diabetic pregnant rats showed significantly reduced levels of glutathione (2.3^a^ ±  0.24), superoxide dismutase (131^a^ ±  5), and catalase (138^a^ ± 7.8) activities in cerebellar homogenates compared to the control group. Daily oral treatment with *M. charantia* significantly decreased MDA (931^ab^ ± 8.4) concentration and significantly increased GSH (3.4^ab^ ±  0.19), SOD (164^ab^ ± 5.2), and CAT (162^ab^ ± 1.5) activities compared to untreated diabetic mothers.

**Fig 3:**
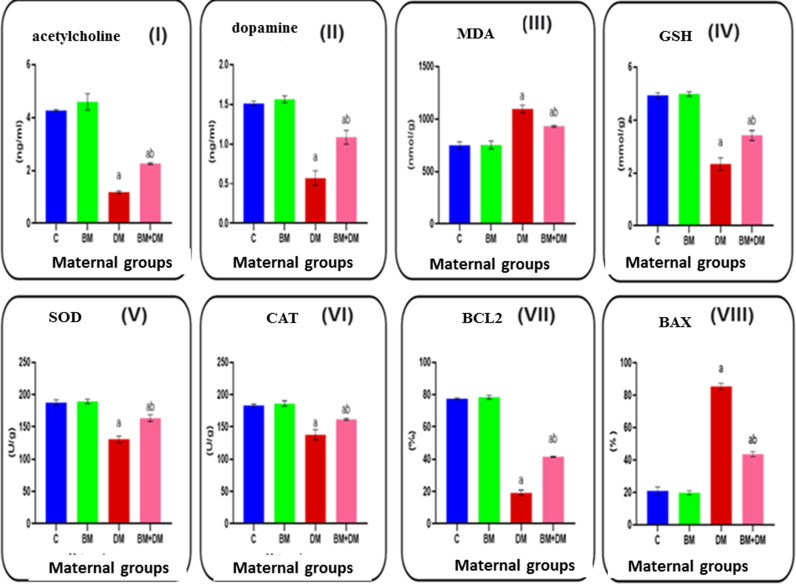
Effect of DM, and/or *M. charantia* on the neurotransmitters levels, acetylcholine (I) and dopamine (II). Oxidative stress markers, Malondialdehyde (III), glutathione (IV), superoxide dismutase (V) and catalase (VI). Apoptotic markers, B-cell lymphoma 2 (VII) and B-cell lymphoma-2(VIII) associated activities in cerebellum tissue homogenate in the control and different treated maternal groups. Values are expressed as means ±  SEM; (n =  6); (a: Significant as compared with the control, *P* < 0.05), (ab: Significant as compared with DM group, *P* <  0.05).

In terms of apoptosis markers, levels of B-cell lymphoma 2 (Bcl-2) were significantly reduced (19^a^ ± 1.6), while BAX levels were significantly increased (85^a^ ± 1.9) in diabetic pregnant rats compared to controls. Treatment with *M. charantia* resulted in a significant increase in Bcl-2 levels (42^ab^ ±  0.34) and a significant decrease in BAX levels (44^ab^ ± 1.5) compared to untreated diabetic mothers (Fig 3).

### 3.4. Histopathological results of cerebellar tissue in mothers

**Fig 4:**
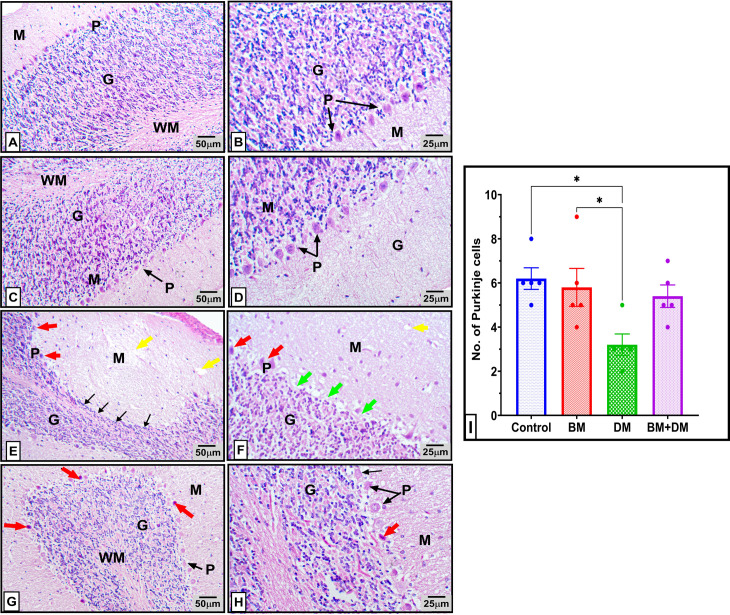
Photomicrographs of H&E-stained cerebellum sections from control and different pregnant mothers: control (A & B) and BM (C & D) groups, showing normal histological structure of the cerebellar cortex three layers; the molecular layer (M) was pale eosinophilic with few cells, the Purkinje cell layer (P) revealed one row of large pear-shaped cells with vesicular nuclei and prominent nucleoli and the granular cell layer (G) exhibited densely packed rounded cells and darkly stained nuclei, and normal white matter (WM). **(E & F)** Cerebellar sections of diabetic mothers showing Vaculation of the neuropil (yellow arrow), a significant loss of Purkinje cells leaving clear remnant spaces (green arrow), a marked pyknosis (red arrow) thin granular layer (black arrow) with marked loss of granule cells. M: molecular layer, P: Purkinje cell layer and G: granular layer. **(G & H)** Cerebellar sections of BM+DM treated group showing overall improvement in the different cerebellar layers The molecular layer (M), the Purkinje cell layer (P), the granular cell layer (G) and the white matter (WM). However, degeneration and pyknosis (red arrow) of a few Purkinje cells were noticed. **(I)**: represents approximate Purkinje cells number in different experimental groups. * p < 0.05. Right panel 100X, left panel 400X.

Histopathological examination of the cerebellum from postnatal control mother rats revealed a normal histological appearance of the cerebellar cortex with its three distinct layers (Fig 4A and 4B). The molecular layer appeared pale with its ubiquitous nerve fibers, scattered stellate, and basket cells, the Purkinje cell layer displayed a single row of large, pear-shaped cells with vesicular nuclei and prominent nucleoli, and the granular cell layer exhibited densely packed, rounded cells with darkly stained nuclei. The medulla was composed of condensed, well-organized nerve fibers interspersed with neuroglial cells. The cerebellar cortex of the *M. charantia* treated mothers (BM group) maintained a normal architecture similar to the control group (Fig 4C and 4D).

However, the cerebellum of diabetic rats displayed moderate atrophy in the neuropil of all cortical layers, along with marked degeneration of Purkinje cells characterized by swollen, pale, and vacuolated neuroplasm (Fig 4E). Significant necrosis of Purkinje cells was observed, accompanied with nuclear pyknosis. In some areas, Purkinje cells were mostly absent, leaving clear empty spaces, or “empty baskets” (Fig 4F). The granular layer appeared irregularly thin, with a marked loss of granule cells.

Conversely, the cerebellum of diabetic rats administered *M. charantia* exhibited predominantly normal histological architecture of the cortex, with only sporadic degenerated apoptotic Purkinje cells with shrunken hyper-eosinophilic, and pyknotic nuclei nuclear pyknosis (Fig 4H and 4I).

### 3.5. Histopathological results of cerebellar tissue in offspring

**Fig 5:**
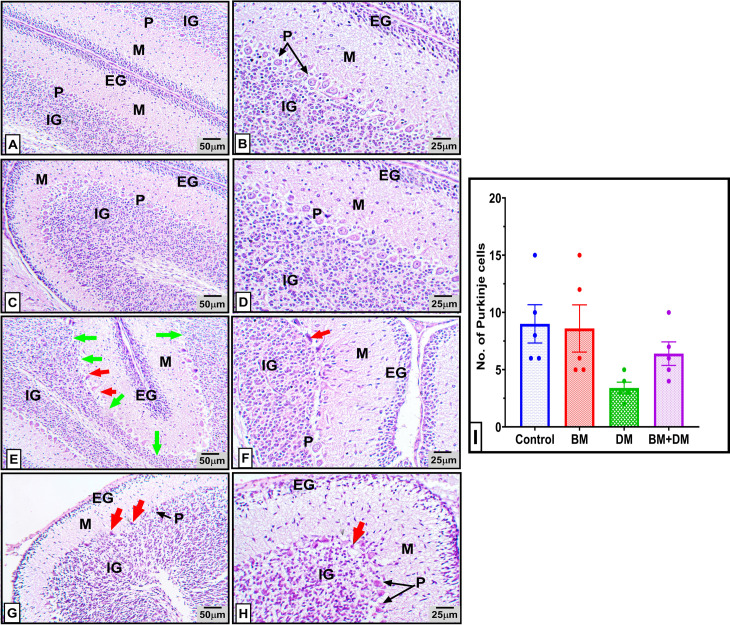
Photomicrographs of H&E-stained cerebellum sections from control and different offspring’s’ groups. Control **(A&B)** and **BM (C&D)** groups, showing the normal four-layered structure of the cerebellar cortex; the external granular layer (EG), the molecular layer (M), the Purkinje cell layer (P) and the internal granular layer (IG). **(E&F)** Cerebellar cortex sections from diabetic mothers showing Focal loss of Purkinje cells leaving empty baskets (green arrow), marked necrosis with hypereosinophilic, shrunken, angular neuroplasm and nuclear pyknosis (red arrow). EG: external granular layer, M: molecular layer, P: Purkinje cell layer and IG: internal granular layer. **(G & H)** Cerebellar cortex sections from DM+BM group showing quite normal external and internal granular cell layer (EG&IG), molecular layer (M), while the Purkinje cell layer (P) displayed a few numbers of degenerated cells with pyknotic nuclei (red arrow). (**I**): represents approximate Purkinje cells number in different experimental groups. Right panel 100X, left panel 400X.

Cerebellar sections from the offspring of control mothers (Fig 5A and 5B) and BM-treated mothers (Fig 5C and 5D) showed the normal four-layered structure of the cerebellar cortex, comprising the external granular layer, molecular layer, Purkinje cell layer, and internal granular layer. In contrast, cerebellar sections from the offspring of diabetic mothers exhibited multifocal loss of Purkinje cells, leaving behind empty baskets. These Purkinje cells showed marked necrosis, characterized by hyper-eosinophilic, and apoptosis characterized with shrunken, angular neuroplasm and nuclear pyknosis (Fig 5E and 5F). Otherwise, the offspring of diabetic mothers treated with *M charantia* (BM+DM group), displayed only mild degeneration and necrosis in a few Purkinje cells, with a slight loss of Purkinje cells (Fig 5G and 5H).

### 3.6. Immunohistochemical results

**Fig 6:**
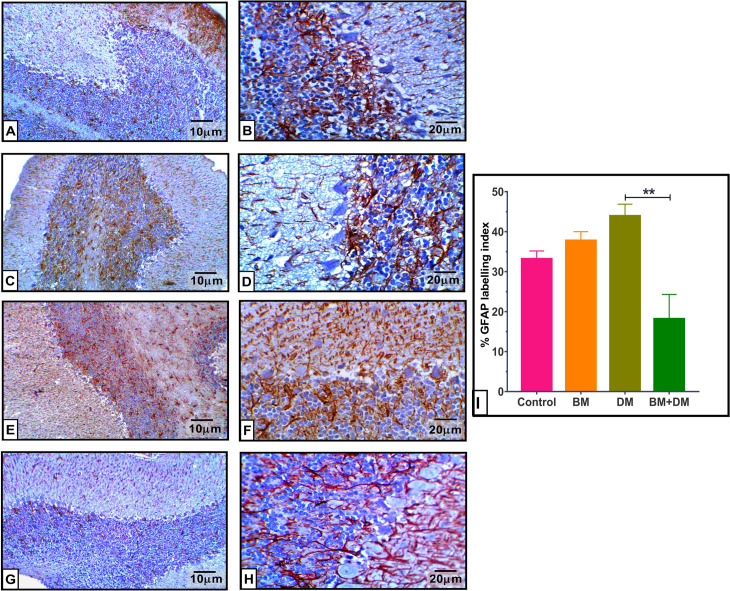
Effect of DM, and/or *M. charantia* on the immuno-expressional level of GFAP protein in maternal cerebellar tissue of different groups. **(A & B)**: The control group revealed tiny astrocytes with short processes and a mild brown stain in the granular and molecular layers, as a typical positive GFAP cells. **(C & D)**: BM group displaying normal positive GFAP cells and small astrocytes with short processes and weak to moderate staining of astroglial cells. **(E & F)**: diabetic group demonstrates a higher prevalence and increased expression of GFAP positive cells and astrocytes. These astrocytes exhibit larger size and greater quantity, with longer processes primarily in the granular layer. Additionally, there are brownish astroglial cells scattered across several levels of the cortex that have a positive reactivity for GFAP. **(G & H)**: BM+DM group revealed a moderate level of GFAP expression, accompanied by a reduction in the quantity and duration of astrocyte processes, in comparison to the diabetic untreated mothers. **(I)**: GFAP labelling index using image j analysis program. left panel 100X, Right panel 400X. The values are expressed as the means±SEM of 5 microscopic fields/tissue samples of GFAP immune-expression, **P < 0.01.

**Fig 7:**
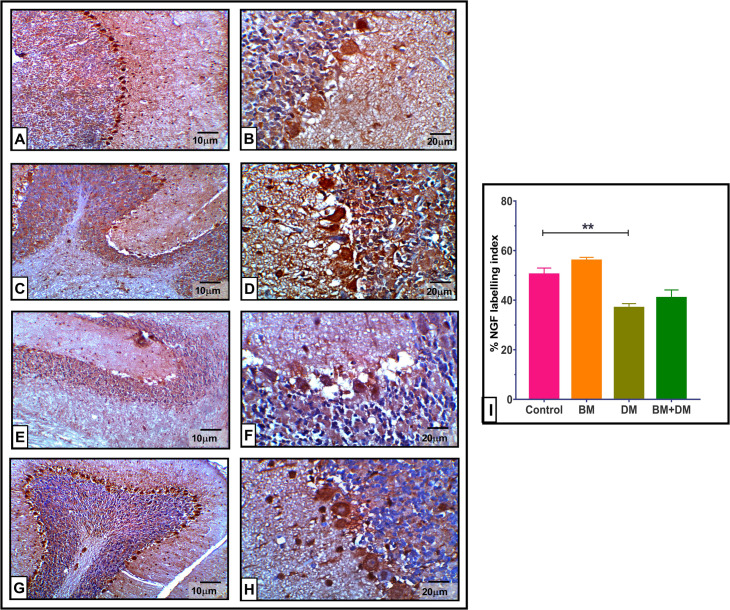
Effect of DM, and/or *M. charantia* on the immuno-expressional level of NGF protein in maternal cerebellar tissue of different groups. Figs **(A & B):** the control group exhibiting typical expression of NGF protein in the molecular layer, Purkinje cells, and molecular layer. Figs **(C & D):** The BM-treated group had normal levels of NGF protein throughout all three layers. Figs **(E & F):** Diabetic mothers have a significant decrease in the expression of NGF protein across all three layers of the cerebellar cortex. Figs **(G & H)**: The BM+DM combination therapy group demonstrated protection against synaptic damage induced by diabetes, with NGF protein levels approaching normalcy. **(I):** NGF labelling index using image j analysis program. left panel 100X, Right panel 400X. The values are expressed as the means±SEM of 5 microscopic fields/tissue samples of NGF immune-expression, **P < 0.01.

The immunohistochemistry analysis of normal cerebellar tissue from mothers treated with *M. charantia* extract (BM group) demonstrated a typical level of GFAP expression in comparison to normal untreated cerebellar tissue. STZ therapy significantly elevated GFAP immuno-expression by 32.46% relative to the control group. However, the administration of *M. charantia* extract to diabetic rats (BM+DM group) resulted in a significant reduction (−58.68%) in GFAP expression compared to untreated diabetic controls, as shown in Fig 6. The neural growth factor (NGF) level in the cerebellar tissue of diabetic rats was remarkably reduced (*P* < 0.01) by 26.80% compared to the control group. Meantime, administering *M. charantia* extract to diabetes mothers resulted in a notable rise in NGF levels by + 10.85% compared to untreated diabetic subjects, as shown in Fig 7.

### 3.7. Ultrastructure of the cerebellum tissue in mothers and their offspring

**Fig 8:**
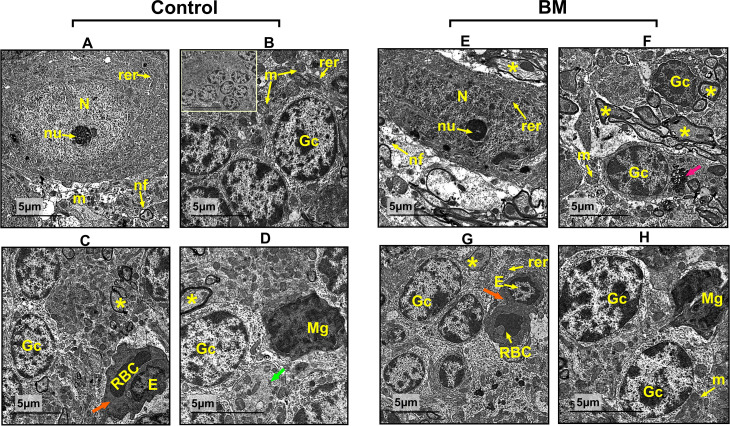
Electron micrographs of cerebellar cortex sections from control mothers (A-D). **(A)**: Displaying typical Purkinje cells characterized by a viable nucleolus (nu) and eukaryotic nucleus (N), the cytoplasm of these cells is replete with rough endoplasmic reticulum (rer), mitochondria (m), and nerve fibers (nf). **(B):** granular layer shows granulocytes (Gc) with cytoplasm rich in rough endoplasmic reticulum (rer) and mitochondria (m). **(C & D):** showing intact neuropil with granulocytes (Gc), microglial cells (Mg), myelinated neural axons (*) forming synapsis, blood capillary (orange arrow) with endothelial cell (E) and red blood corpuscle (RBC) (green arrow) represents neuropil multivesicular body. **(E–H)** Cerebellar cortex sections from BM mothers showing, **(E):** exhibit normal Purkinje cells with normal eukaryotic nucleus (N) and noticeable nucleolus (nu), cytoplasm contains rough endoplasmic reticulum (rer) and mitochondria (m) and nerve fibers (nf) around it. **(F):** The granular layer exhibits granulocytes (Gc) characterized by cytoplasm with abundant mitochondria (m), neural synapsis and neuropil multivesicular body (colored arrow). **(G & H):** The image displays an undamaged neuropil with granulocytes (Gc), microglial cells (Mg), mitochondria (m), myelinated neural axons establishing synapses, a blood capillary (shown by an orange arrow) with an endothelial cell (E), and a red blood corpuscle (RBC).

Examination of the cerebellar cortex from control (Fig 8A–8D) and BM-treated mothers (Fig 8E–8H) revealed an intact pyriform cell with a distinct eukaryotic nucleus and a prominent electron dense nucleolus. The cytoplasm was abundant in rough endoplasmic reticulum (RER) and secretory granules, as well as both rounded and elongated mitochondria. The granular layer exhibited typical granulocytes with predominantly peripheral and minimal central euchromatin, centric heterochromatin, and well-defined nucleoli. along with neuropil containing granulocytes, microglial cells, and myelinated neural axons forming pre- and post-synapses. The presence of blood capillaries with endothelial cells and red blood corpuscles was also observed, representing the multivesicular bodies within the neuropil.

**Fig 9:**
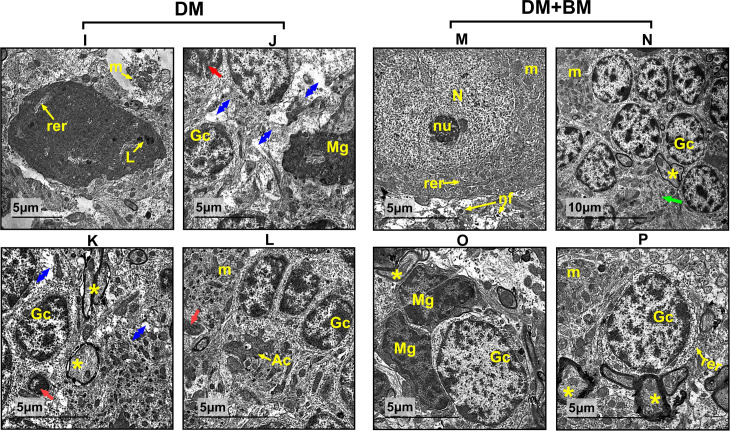
Electron micrographs of cerebellar cortex sections from diabetic mothers (I–L). Cerebellar cortex sections from diabetic mothers showing, **(I):** reveals apoptotic Purkinje cell with indefinite nucleus and dilation in (rer) and dark lysosomal patches **(L)**, swallowed mitochondria (m) could be noticed. **(G–L):** showing granular layer suffering from neuropil degenerative changes (blue arrow), abnormal granulocytes (Gc) and microglial cells (Mg), apoptotic bodied with pyknotic nuclei (red arrows) and dilated neural axons (*). Astrocyte of fibre-type (Ac) could be distinguished. **(M–P)** Cerebellar cortex sections from DM+BM group showing, **(M):** Combined treatment shows quite normal Purkinje cell with a typical eukaryotic nucleus (N) and a discernible nucleolus (nu), its cytoplasm is surrounded by nerve fibers (nf), mitochondria (m), and rough endoplasmic reticulum (rer). **(N–P):** showing the granular layer with a complete neuropil comprising granulocytes (Gc), microglial cells (Mg), myelinated neural axons (*) that have formed synapses, (green arrow) Denotes the multivesicular body of the neuropil.

In diabetic mothers (Fig 9I–9L), Purkinje cells appeared apoptotic, with indefinite nuclei, dark lysosomal patches, and swollen mitochondria. The granular layer showed degenerative changes in the neuropil, including abnormal granulocytes, microglial cells, apoptotic bodies with pyknotic nuclei, and dilated neural axons.

In the diabetic mothers treated with *M. charantia* (BM+DM group) (Fig 9M–9P), the cerebellar cortex displayed relatively normal Purkinje cells, with well-defined eukaryotic nuclei and discernible nucleoli. The cytoplasm contained normal rough endoplasmic reticulum, mitochondria, and surrounding nerve fibers. The granular layer appeared complete, containing granulocytes, microglial cells, and myelinated neural axons forming synapses, reflecting a healthy multivesicular body in the neuropil.

**Fig 10:**
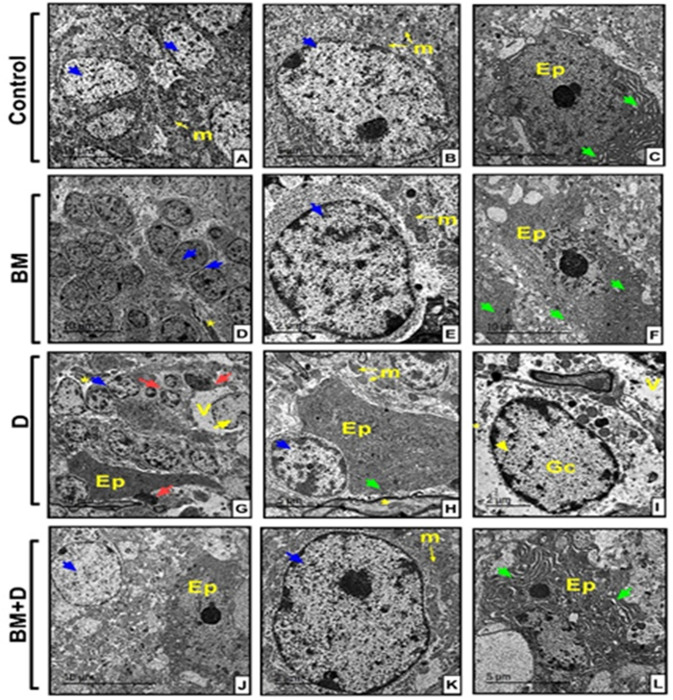
Electron micrographs of cerebellar cortex sections from different offspring’s groups. The cerebellar cortex of both control **(A–C)** and BM-extract treated offspring **(D–F)** revealed intact neurons (blue arrow) with a distinct eukaryotic nucleus and prominent electron dense nucleoli, surrounded by numerous mitochondria (m). Clearly, there were neural axons (*) and active Ependymal cells (Ep) with distinct nucleoli and microvilli (green arrow) scattered throughout the neuropil. **(G–I)** Cerebellar fetal cortex from diabetic mothers depicts affected neurons with atypical nuclei (yellow arrow) or apoptotic with pyknotic nuclei (red arrow), neuropil showed vacuolated (v) with a fewer number of mitochondria (m). Neural axons showed not affected (*), while Ependymal cells (Ep) showed irregular with fewer microvilli (green arrow) in-between neurons (blue arrow). **(J–L)** Cerebellar fetal cortex from diabetic mothers treated with BM-extract revealed remarkable protection of the fetal cerebellar cortex characterized by an abundance of normal neurons displaying active nuclei and well-defined nucleoli (shown by the blue arrow), which were surrounded by a multitude of mitochondria (m). Evidently, neuropil included neuronal axons (*) and active Ependymal cells (Ep) exhibiting discernible nucleoli and microvilli (shown by the green arrow).

Further examination of the cerebellar cortex in offspring from control mothers (Fig 10A–10C) and BM-treated mothers (Fig 10D–10F) revealed intact neurons. Neural axons and active ependymal cells with distinct nucleoli and microvilli were scattered throughout the neuropil. In contrast, the fetal cerebellar cortex from untreated diabetic mothers (Fig 10G–10I) showed affected neurons with atypical or apoptotic nuclei (pyknotic). The neuropil appeared vacuolated, with a reduced number of mitochondria. While the neural axons were relatively unaffected, the ependymal cells were irregular and had fewer microvilli. Treatment of diabetic mothers with BM extract (Fig 10J–10L) offered remarkable protection to the fetal cerebellar cortex, which was characterized by an abundance of normal neurons with active nuclei and well-defined nucleoli. The cytoplasm was rich in mitochondria, and the neuropil contained healthy neuronal axons and active ependymal cells with discernible nucleoli and microvilli.

## 4. Discussion

The use of medicinal herbs in the treatment of diabetes is gaining attention for its potential to decrease the risk of birth defects in cases of maternal diabetes. Given this, identifying a safe and effective diabetes treatment is a priority for researchers [4]. Bitter melon (*M charantia*) is widely consumed as a vegetable in several Asian countries [27]. Experimentally, the fruit extract of bitter melon has been shown to possess potent antioxidant, hypoglycemic, and therapeutic effects, particularly in the context of diabetes and obesity-related metabolic dysfunction [28].

The several bioactive compounds found in *M. charantia,* including vicine, charantin, glycosides, karavilosides, polypeptide-p, and plant insulin, are responsible for its anti-diabetic actions. Triterpene, protein, steroids, alkaloids, inorganic, lipid, and phenolic compounds are all members of the large family of phytochemicals that includes these beneficial substances. There have been reports of *M. charantia’s* anti-diabetic effects in both type 1 and type 2 diabetes. According to **Liu et al.** [29], and **Xu et al.** [30] in normal animals, diabetic models caused by streptozotocin, alloxan-induced diabetics and diabetes genetic models, every morphological component of *M. charantia* showed hypoglycemic action. *M. charantia* has demonstrated promising effects in avoiding diabetes mellitus and delaying the progression of diabetic sequelae, such as neuropathy, gastroparesis, nephropathy, waterfall, and insulin blockage, in experimental animal models.

Beta-momorcharin compound in bitter melon, may induce abortifacient effects in animal studies. This abortive effects noted in animals are typically linked to purified compounds or high doses, which exceed normal dietary consumption. In the current study, we carefully selected dose based on existing literature to avoid potential toxicity or abortifacient effects. The doses used were within ranges considered safe and effective for managing diabetes without reported adverse reproductive effects [4,31].

In the current study, there was considerably fewer neonates’ number in the diabetes group than in the other categories. These findings concurred with those of **Wang et al.** [32], who found that in cases of maternal diabetes, elevated intrauterine glucose levels are caused by the glucose transporter protein GLUT1 transferring significant amounts of maternal glucose across the placental barrier into the fetal circulation. This process, which results in fetal death and fewer neonates, is mainly caused by an imbalance in cellular apoptosis and proliferation as well as an increase in oxidative stress. Nonetheless, the number of a live offsprings increased after treatment with bitter melon extract, which is consistent with **Elnahas et al.** [4], who discovered that the bitter melon fruit extract had strong antioxidant, hypoglycemic, and possible therapeutic effects against metabolic dysfunction linked to diabetes and obesity.

In the current study, there was a notable decrease in newborn weight in the DM group. **Choudhury et al.** [33], explained that numerous environmental factors are linked to an increased risk of fetal deformity and low weight, and the maternal diabetes environment during embryogenesis has a significant impact on fetal development. In contrast to the diabetes group, the MC and DM+MC groups showed an increase in newborn weight. According to **Xu et al.** [34], bitter melon extract alters mitochondrial activity, reduces oxidative stress and inflammation, inhibits lipid accumulation during the development of liver fat, and regulates the activation of apoptosis [35].

The diabetic group showed high and substantial reductions in newborn length, due to diabetic embryopathy, which is characterised by fetal abnormalities with preexisting type 1 or type 2 diabetes. The risk of congenital malformations is up to five times higher in pregnancies with diabetes. During the first 10 weeks of pregnancy, early organogenesis results in abnormalities [36]. There is no question that the intrauterine environment experiences oxidative stress due to the production of reactive oxygen species (ROS) during hyperglycemia during pregnancy. This condition may cause harm to the embryo, placenta, fetus, and offspring [37]. In contrast to the diabetes group, the MC and DM+MC groups demonstrated improvements in neonatal length. This is because, in addition to the phenolic components, the polysaccharides in bitter melon extract also have antioxidant qualities [35].

In the present study, we observed a significant increase in serum glucose, HOMA-IR, insulin, and leptin levels in diabetic pregnant rats compared to control mothers. Similar results were reported by **Elnahas et al.** [4], **Goya et al.** [38] and **Rouhi et al**. [39]. That’s may be due to the cytotoxic effect of streptozotocin (STZ) on pancreatic β-cells likely contributed to hyperglycemia by reducing β-cell function. **Seong et al.** [40] also proposed that insulin resistance (IR) could result from the compensatory increase in insulin production by the remaining β-cells. Additionally, **Otani** [41] suggested that increased oxidative stress contributes to the development of IR by promoting inflammation. Insulin resistance in the liver leads to altered glucose and lipid metabolism, which worsens hyperglycemia [42,43].

Our results demonstrated that treatment of diabetic rats with *M. charantia* extract significantly lowered serum glucose, HOMA-IR, insulin, and leptin levels. This effect may be attributed to the inhibition of α-amylase and α-glucosidase by compounds in *M. charantia*, as reported by **Wang et al.** [10], which suggests improved insulin sensitivity and postprandial glucose regulation [44,45].

Furthermore, our data revealed a significant reduction in QUICKI, GLUT4, and AMPK levels in diabetic mothers compared to controls. The reduction in these molecules contributes to a cycle of insulin resistance, impaired glucose transport, and disrupted cellular energy regulation, exacerbating the symptoms and complications of diabetes [46].

Interestingly, treatment with *M. charantia* significantly increased these markers in diabetic mothers, consistent with studies by **Poovitha and Parani** [38], **and Han et al.** [47]. The extract may enhance GLUT4 translocation in muscle cells [48] and activate AMPK through triterpenoids present in the bitter melon, improving insulin sensitivity. Bitter melon may be a useful supplement to promote energy balance and improve glucose homeostasis, potentially reducing some of the insulin resistance and glucose transport problems typical in diabetes. This is because it can improve QUICKI, GLUT4 and AMPK pathways [49].

Additionally, maternal diabetes led to reduced acetylcholine and dopamine levels, corroborating findings by **Kroner** [50] **and Iuliis et al.** [51]. The link between hyperglycemia, IR, and impaired ACh production is well-established [52]. Dopamine’s regulatory role in insulin secretion via type 2 receptors further complicates this dynamic. In conclusion, insulin resistance, oxidative stress, and inflammation all contribute to the reduction of ACh and dopamine in diabetes. This affects mood and cognition, increasing the risk of mood disorders and cognitive impairment in diabetics [53].

Bitter melon extract treatment restored ACh and dopamine levels, as previously reported by **Kavitha et al.** [54]. Antioxidants and anti-inflammatory substances included in bitter melon extract may shield neurones from oxidative damage and inflammation, two things that lower ACh levels in diabetics. Bitter melon can help sustain or even restore ACh synthesis by lowering oxidative damage and promoting the health of cholinergic neurones. Furthermore, the ability of bitter melon to increase insulin sensitivity and glucose metabolism may increase the brain’s energy supply, promoting the synthesis of ACh and cognitive performance [55]. Furthermore, the antioxidant activity of the bitter melon extract might shield dopaminergic neurones from harm, assisting in the restoration of dopamine levels and thus enhancing motivation and mood [56].

Oxidative stress markers in cerebellar tissues, such as reduced GSH, SOD, and CAT activities, alongside elevated MDA levels, were observed in diabetic mothers. These findings are consistent with those reported by **Chen et al.** [57] and **Saravanan and Ponmurugan** [58], who linked oxidative stress to mitochondrial dysfunction and apoptosis in diabetic conditions. Bitter melon treatment restored antioxidant enzyme activity and reduced MDA levels, supporting previous studies that demonstrate the extract’s ability to mitigate oxidative stress and promote mitochondrial function. All these results highlight an imbalance between ROS production and antioxidant defense in diabetes. This oxidative stress contributes to complications such as neuropathy disease and nephropathy [59].

Bioactive substances found in bitter melon, including triterpenoids, phenolic acids, and flavonoids, have potent anti-free radical capabilities. These elements lower MDA levels by neutralising reactive oxygen species (ROS) before they have a chance to start lipid peroxidation [60] By stimulating the transcription factor Nrf2 (nuclear factor erythroid 2–related factor 2), which increases the expression of genes involved in GSH manufacture, including glutamate-cysteine ligase, *M. charantia* increases the synthesis of GSH. According to **Barakat** [61], its components, including phenolic compounds, cucurbitane-type triterpenoids, and saponins, may also suppress ROS and GSH-consuming enzymes, maintaining intracellular GSH levels. Due to the presence of flavonoid compounds, vitamin C, and polypeptide-p, which play a role in Nrf2 activation and promote SOD transcription, *M. charantia* has been demonstrated to activate SOD activity through elevation of antioxidant enzyme expression. *M. charantia’s* phenolic acids, vitamin E, and saponins can increase CAT activity, most likely via activating Nrf2, which raises the expression of the CAT gene. Furthermore, its antioxidant components indirectly boost CAT function by directly lowering the ROS burden [62].

The Nrf2 pathway is activated by the bioactive components of *M. charantia* supplements, such as flavonoids and phenolic acids. This leads to an upregulation of genes that encode antioxidant enzymes such as SOD, CAT, and enzymes involved in the manufacture of GSH.*M. charantia* contains compounds such flavonoids, triterpenoids, and vitamins C and E that can directly neutralise ROS, lowering MDA levels and lipid peroxidation. Polypeptide-p and other bitter melon components reduce the generation of ROS by lowering hyperglycemia, which indirectly improves SOD and CAT activity and preserves GSH [63].

Apoptotic markers were also elevated in diabetic mothers, as indicated by increased Bax levels and decreased Bcl2 expression, similar to findings by **Wu et al.** [65] **and Yang et al.** [64]. The increase in Bax and decrease in Bcl-2 levels in diabetes create a pro-apoptotic environment, leading to higher rates of cell death. This imbalance contributes to the loss of functional cells in the nervous tissue. Apoptosis may also be induced by reactive oxygen species (ROS). Through the mitochondrial route, oxidative stress brought on by excessive ROS can cause apoptosis by damaging cellular components [65,66].

Bitter melon treatment reversed these changes, reducing the Bax/Bcl2 ratio, as reported by **Moustafa et al.** [67], demonstrating its protective effects against apoptosis. This extract could help to reduce apoptosis in diabetic tissues, potentially slowing the progression of diabetes-related cell and tissue damage. This protective effect may contribute to improved outcomes in managing diabetic complications. Apoptosis also may be triggered by compounds in *M. charantia* by modifications in the permeability of the mitochondrial membrane and the release of pro-apoptotic proteins such as cytochrome c, which activates caspase-9, which in turn activates downstream caspases, including caspase-3, which carries out apoptosis. Some investigations using *M. charantia* extract have shown that the balance is tipped towards apoptosis due to the upregulation of pro-apoptotic proteins like Bax and the downregulation of anti-apoptotic proteins like Bcl-2 [68].

Moreover, we noted a significant increase in GFAP-positive astrocytes in diabetic mothers, consistent with **Zare et al.** [69]. Hyperglycemia may exacerbate astrocyte activation, contributing to neuroinflammation and reactive gliosis [70,71]. The moderated GFAP expression observed after bitter melon treatment suggests that the extract deactivates astrocytes, reducing neuroinflammation [72–75]. Nerve growth factor (NGF) expression was reduced in diabetic mothers, aligning with findings by **Oza and Kulkarni** [76], who linked diminished NGF to neuronal degeneration. Bitter melon treatment protected against NGF impairment, suggesting neuroprotective effects.

*M. charantia* contains compounds that have demonstrated anti-inflammatory properties, especially triterpenoids, which may alter GFAP signalling, a marker for astrocyte activation. This may lower neuroinflammation by influencing GFAP expression levels. These substances may lessen GFAP overexpression by suppressing pro-inflammatory cytokines (TNF-α and IL-1β), preserving glial homeostasis [77]. For neurones to survive and be maintained, nerve growth factor (NGF) is essential. Phenolic acids and flavonoids, two bioactive substances found in *M. charantia,* have antioxidant qualities that can increase the expression of NGF. These substances have the ability to lessen oxidative stress, which fosters an atmosphere that is favourable for the synthesis of neurotrophins, including NGF. According to some research, the flavonoids and saponins found in bitter melon may activate ERK1/2 and Akt, two pathways that can result in the release of NGF [78].

Histological analyses of the cerebellum from diabetic mothers and their offspring’s revealed significant degeneration of Purkinje cells and granular cell layers. Similar findings have been reported by **Selim and Selim** [79]**, and Razi et al.** [80], indicating that hyperglycemia disrupts neural development and promotes oxidative stress, ultimately leading to cell death in the CNS [81–86]. Bitter melon supplementation mitigated these effects, preserving cerebellar architecture, likely due to its antioxidant and neuroprotective properties [87–89].

At the ultrastructural level, cerebellar tissue from diabetic mothers and their offspring’s exhibited significant apoptosis in Purkinje cells, with evidence of mitochondrial dysfunction, endoplasmic reticulum dilation, and abnormal microglial cells. These findings are in line with **Niyomchan et al.** [90] **and Shalaby et al.** [91], who documented similar neuropathological changes in diabetic conditions. The neuroprotective effect of bitter melon may be attributed to its ability to modulate oxidative stress and inflammation at the cellular level. The intracellular portions of the neuron may absorb excess blood glucose, which would raise the neuron’s glucose levels by about four times [92,93]. Increased oxidative stress, which produces ROS and lipid peroxidation, was triggered by a high glucose level in the cell [1]. This often results in increased mortality of neural cells and oxidation of proteins, DNA damage, and peroxidation of the lipids in plasma membranes [94]. In addition to glucose auto-oxidation, low glutathione levels in tissues, and decreased antioxidant enzyme activity, the oxidative stress associated with diabetes mellitus increases the vulnerability of brain tissue to oxidative damage.

On the other hand, the cerebellum of diabetic pregnant rats and their offspring treated with *M. charantia* revealed remarkable protection of the mothers and fetal cerebellar cortex characterized by an abundance of normal neurons displaying active nuclei and well-defined nucleoli, which were surrounded by a multitude of mitochondria. Evidently, the neuropil included neuronal axons and active Ependymal cells exhibiting discernible nucleoli and microvilli. This improvement in the tissue structure may due to that *M. charantia* polysaccharides (MCPs) might have functions such as anti-diabetes, neuroprotection, anti-inflammation, anti-oxidation and lipid-lowering [95].

## 5. Conclusion

*M. charantia* demonstrates potent neuroprotective, and antioxidant, improving cerebellar pathology in diabetic mothers and their offspring. These effects are likely mediated through the modulation of oxidative stress, apoptosis, and inflammation, preserving the health of neural tissues. Future studies are warranted to further explore these protective mechanisms and confirm the therapeutic potential of *M. charantia* in diabetic pregnant mothers and their offspring.

## Supporting information

S1 File Raw data. (PDF)
