# Supplementary material for: Restorative effects of Momordica charantia extract on cerebellar GFAP and NGF expression in pregnant diabetic rats and their offspring
Source: PLoS One. 2025 Apr 4;20(4):e0321022. doi: 10.1371/journal.pone.0321022 (PMC11970674; doi:10.1371/journal.pone.0321022)
Supplement: S1 File — (PDF) [file pone.0321022.s001.pdf]

Table (23): BAX level (%) in cerebrum of control and different maternal groups.

Maternal groups		C	BM	DM	BM+DM
Mean		21	20	85[a]	44[ab]
±SE		±2.5	±1.2	±1.9	±1.5
% of change	*		-4.76	304.76	109.52
	**				-48.24

Results are presented as means ±SE and % of change.
(n=6 for each group).
a, b significant changes at $p<0.05$.
a: significant as compared to control.
b: significant as compared to DM group.
(*): % of change related to control group.
(**): % of change related to DM group.
BM: Bitter melon, DM: Diabetes Mellitus.
.

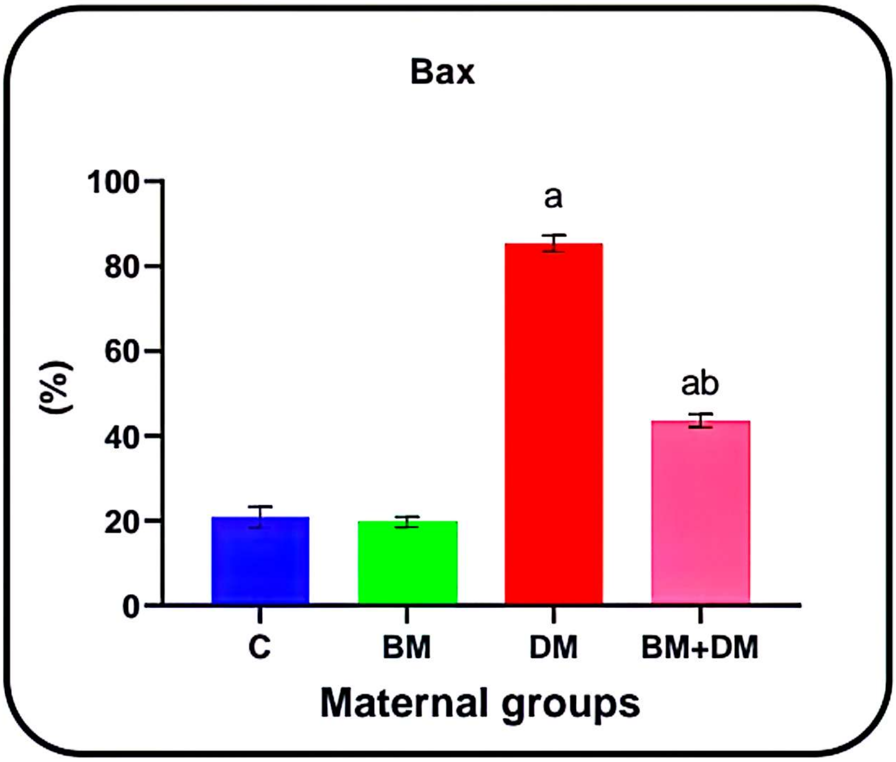

Figure (23 a): BAX level (%) in cerebrum of control and different maternal groups.

CamScanner

Table (22): BCL2 level (%) in cerebrum of control and different maternal groups.

Maternal groups		C	BM	DM	BM+DM
Mean		78	79	19[a]	42[ab]
±SE		±0.52	±1.2	±1.6	±0.34
% of change	*		1.28	-75.64	-46.15
	**				121.05

Results are presented as means ±SE and % of change.
(n=6 for each group).
a, b significant changes at $p<0.05$.
a: significant as compared to control.
b: significant as compared to DM group.
(*): % of change related to control group.
(**): % of change related to DM group.
BM: Bitter melon, DM: Diabetes Mellitus.
.

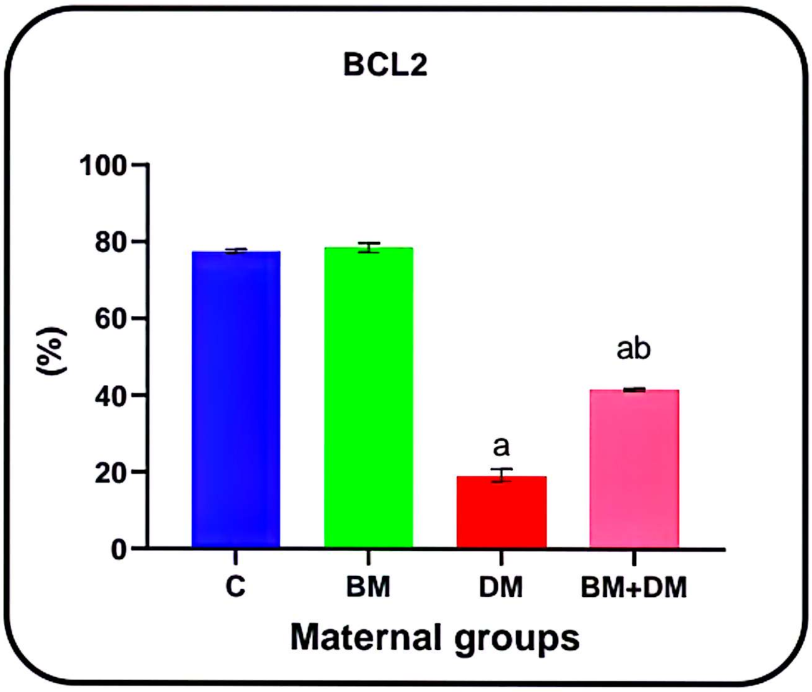

Figure (22 a): BCL2 level (%)in cerebrum of control and different maternal groups.

CamScanner

Table (21): Catalase activity (U/g) in cerebrum of control and different maternal groups.

Maternal groups		C	BM	DM	BM+DM
Mean		183	187	138[a]	162[ab]
±SE		±2.1	±4.1	±7.8	±1.5
% of change	*		2.19	-24.59	-11.48
	**				17.39

Results are presented as means ±SE and % of change.
(n=6 for each group).
a, b significant changes at $p<0.05$.
a: significant as compared to control.
b: significant as compared to DM group.
(*): % of change related to control group.
(**): % of change related to DM group.
BM: Bitter melon, DM: Diabetes Mellitus.
.

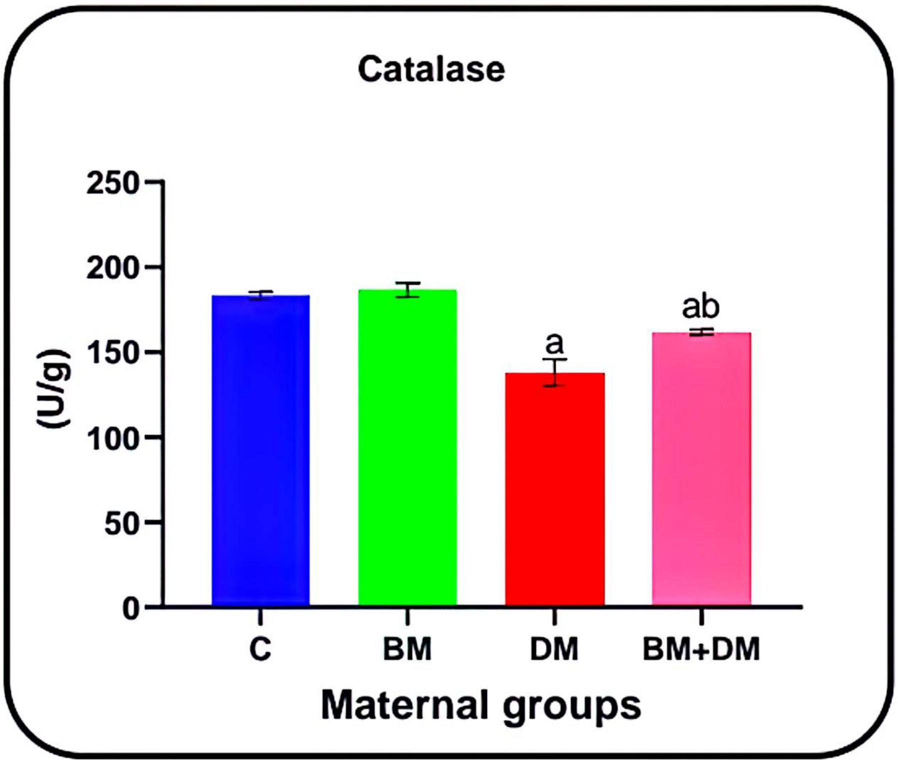

Figure (21): Catalase activity (U/g) in cerebrum of control and different maternal groups.

CamScanner

Table (20): Superoxide dismutase (SOD) activity (U/g) in cerebrum of control and different maternal groups.

Maternal groups		C	BM	DM	BM+DM
Mean		188	190	131[a]	164[ab]
±SE		±4.3	±3.3	±5	±5.2
% of change	*		1.06	-30.32	-12.77
	**				25.19

Results are presented as means ±SE and % of change.
(n=6 for each group).
a, b significant changes at $p<0.05$.
a: significant as compared to control.
b: significant as compared to DM group.
(*): % of change related to control group.
(**): % of change related to DM group.
BM: Bitter melon, DM: Diabetes Mellitus.
.

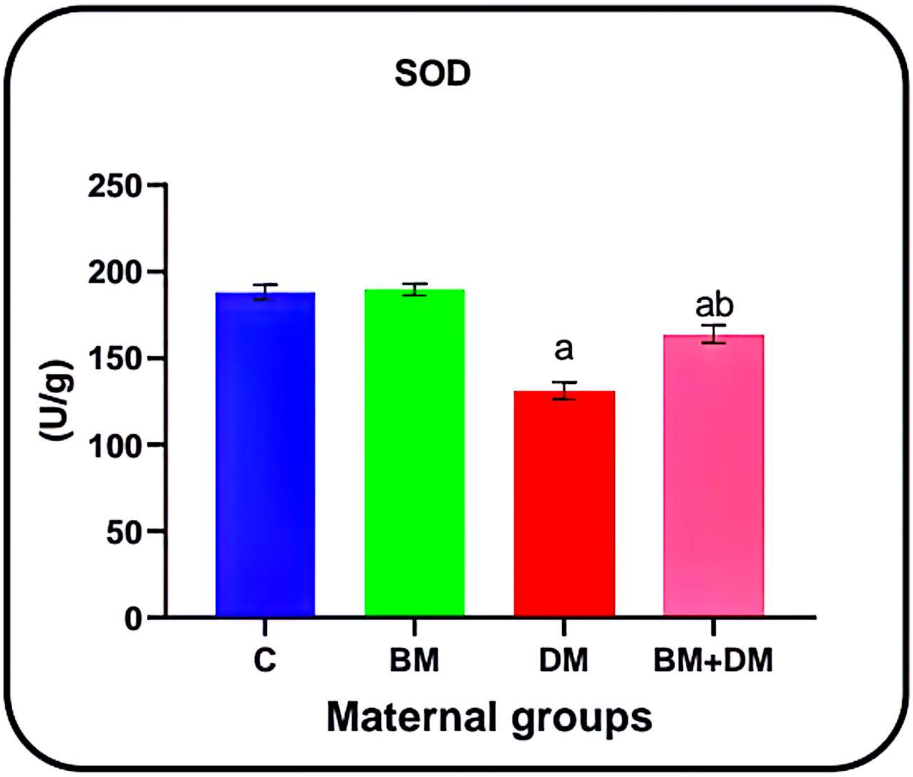

Figure (20): Superoxide dismutase (SOD) activity (U/g) in cerebrum of control and different maternal groups.

CamScanner

Table (19): Glutathione reduced (GSH) (mmol/g) in cerebrum of control and different maternal groups.

Maternal groups		C	BM	DM	BM+DM
Mean		4.9	5	2.3[a]	3.4[ab]
±SE		±0.1	±0.084	±0.24	±0.19
% of change	*		2.04	-53.06	-30.61
	**				47.83

Results are presented as means ±SE and % of change.
(n=6 for each group).
a, b significant changes at p<0.05.
a: significant as compared to control.
b: significant as compared to DM group.
(*): % of change related to control group.
(**): % of change related to DM group.
BM: Bitter melon, DM: Diabetes Mellitus.
.

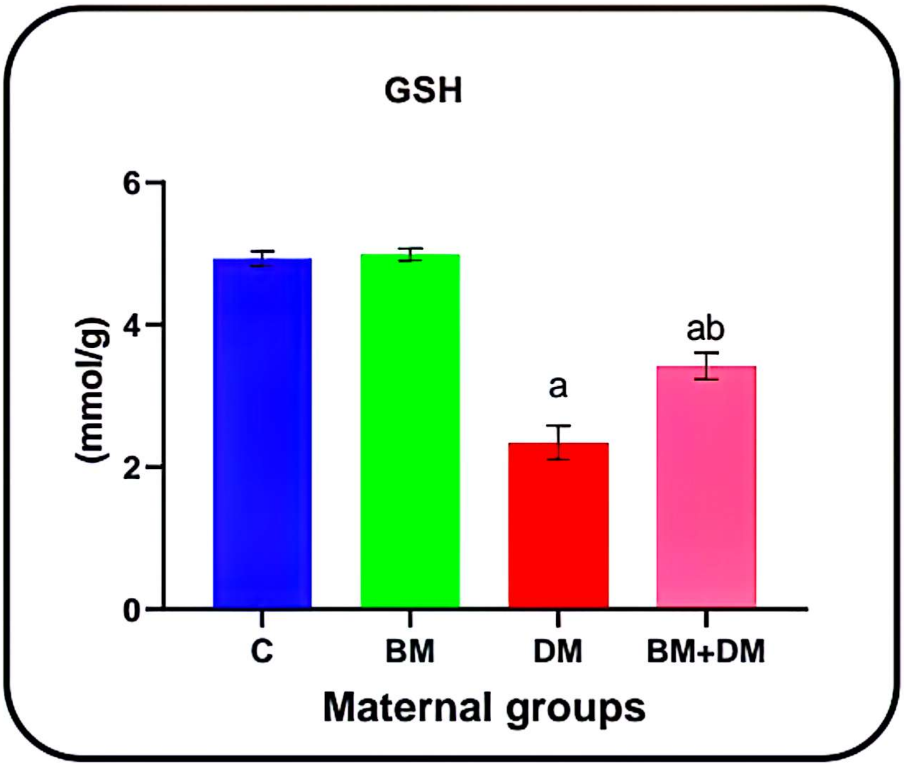

Figure (19): Glutathione reduced (GSH) (mmol/g) in cerebrum of control and different maternal groups.

CamScanner

Table (18): Malondialdehyde (MDA) concentration (nmol/g) in cerebrum of control and different maternal groups.

Maternal groups		C	BM	DM	BM+DM
Mean		750	753	1096[a]	931[ab]
±SE		±31	±35	±37	±8.4
% of change	*		0.40	46.13	24.13
	**				-15.05

Results are presented as means ±SE and % of change.
(n=6 for each group).
a, b significant changes at $p<0.05$.
a: significant as compared to control.
b: significant as compared to DM group.
(*): % of change related to control group.
(**): % of change related to DM group.
BM: Bitter melon, DM: Diabetes Mellitus.
.

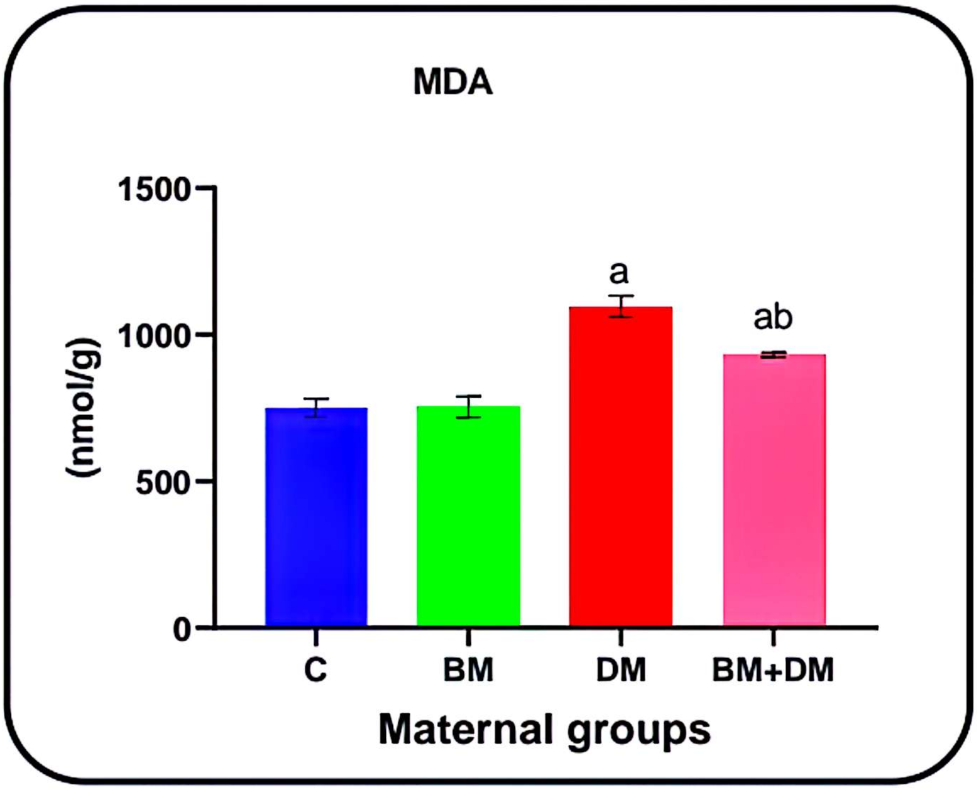

Figure (18): Malondialdehyde (MDA) concentration (nmol/g) in cerebrum of control and different maternal groups.

CamScanner

Table (17): Serum Leptin level (ng/ml) in control and different maternal groups.

Maternal groups		C	BM	DM	BM+DM
Mean		20	19	36[a]	27[ab]
±SE		±1.20	±1.50	±2.10	±1.20
% of change	*		-5	80	35
	**				-25

Results are presented as means ±SE and % of change.
(n=6 for each group).
a, b significant changes at $p<0.05$.
a: significant as compared to control.
b: significant as compared to DM group.
(*): % of change related to control group.
(**): % of change related to DM group.
BM: Bitter melon, DM: Diabetes Mellitus.

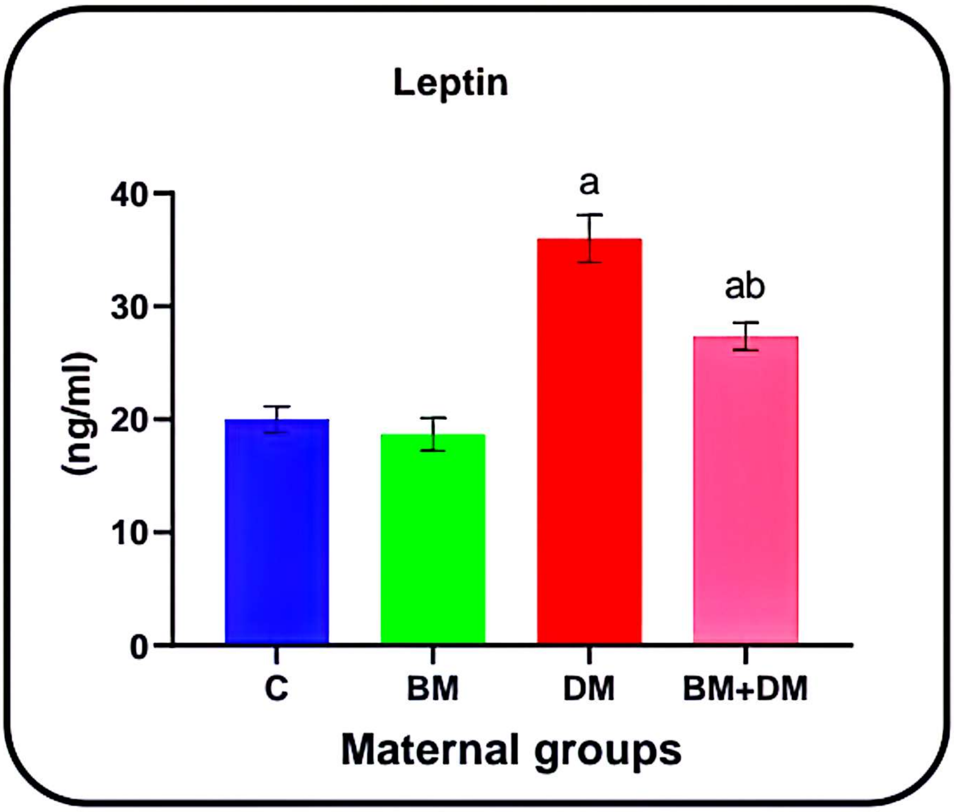

Figure (17): Serum Leptin level (ng/ml) in control and different maternal groups.

CamScanner

Table (16): Serum GLUT4 level (ng/ml) in control and different maternal groups.

Maternal groups		C	BM	DM	BM+DM
Mean		0.37	0.41	0.1[a]	0.23[ab]
±SE		±0.027	±0.041	±0.019	±0.0072
% of change	*		10.81	-72.97	-37.84
	**				130.00

Results are presented as means ±SE and % of change.
(n=6 for each group).
a, b significant changes at $p<0.05$.
a: significant as compared to control.
b: significant as compared to DM group.
(*): % of change related to control group.
(**): % of change related to DM group.
BM: Bitter melon, DM: Diabetes Mellitus.

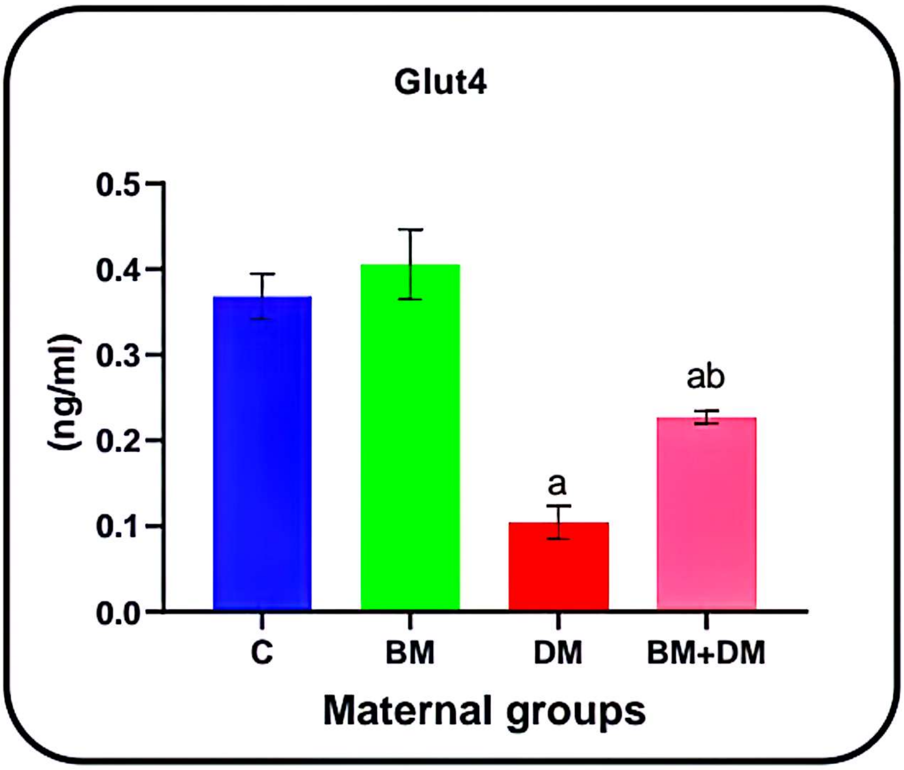

Figure (16): Serum GLUT4 level (ng/ml) in control and different maternal groups.

CamScanner

Table (15): Serum AMPK (ng/ml) in control and different maternal groups.

Maternal groups		C	BM	DM	BM+DM
Mean		15	16	9.5	12
±SE		±0.22	±0.42	±0.32	±0.56
% of change	*		6.67	-36.67	-20.00
	**				26.32

Results are presented as means ±SE and % of change.
(n=6 for each group).
a, b significant changes at $p<0.05$.
a: significant as compared to control.
b: significant as compared to DM group.
(*): % of change related to control group.
(**): % of change related to DM group.
BM: Bitter melon, DM: Diabetes Mellitus.

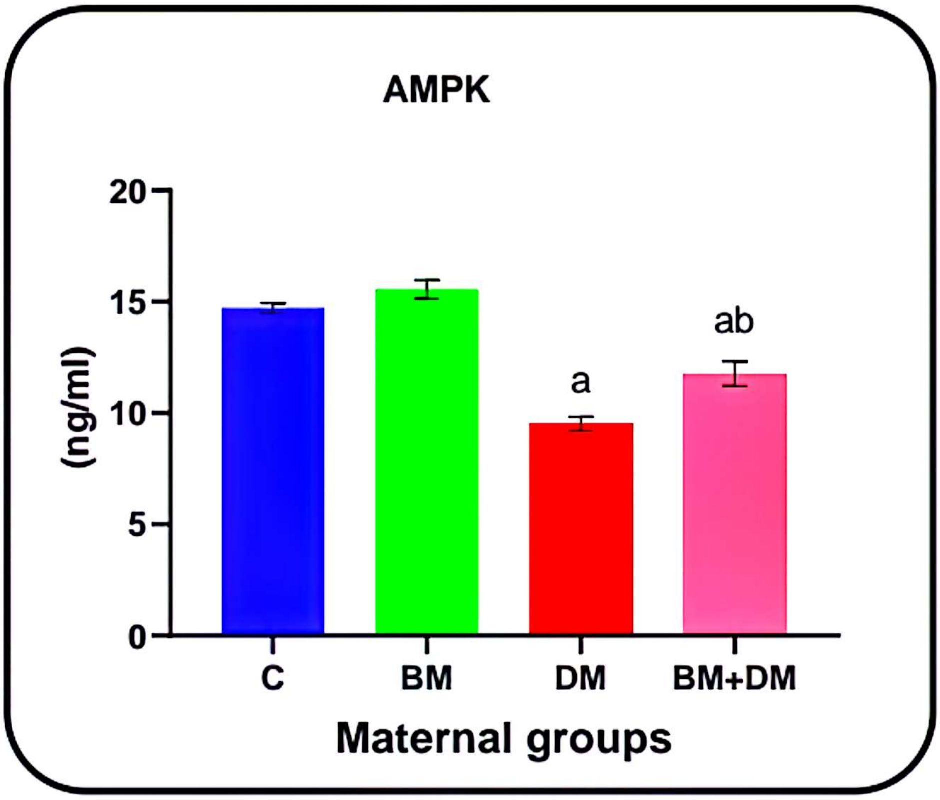

Figure (15): Serum AMPK (ng/ml) in control and different maternal groups.

CamScanner

Table (14): Serum Dopamine level (ng/ml) in control and different maternal groups.

Maternal groups		C	BM	DM	BM+DM
Mean		1.5	1.6	0.57[a]	1.1[ab]
±SE		±0.028	±0.042	±0.092	±0.087
% of change	*		6.67	-62.00	-26.67
	**				92.98

Results are presented as means ±SE and % of change.
(n=6 for each group).
a, b significant changes at $p<0.05$.
a: significant as compared to control.
b: significant as compared to DM group.
(*): % of change related to control group.
(**): % of change related to DM group.
BM: Bitter melon, DM: Diabetes Mellitus.

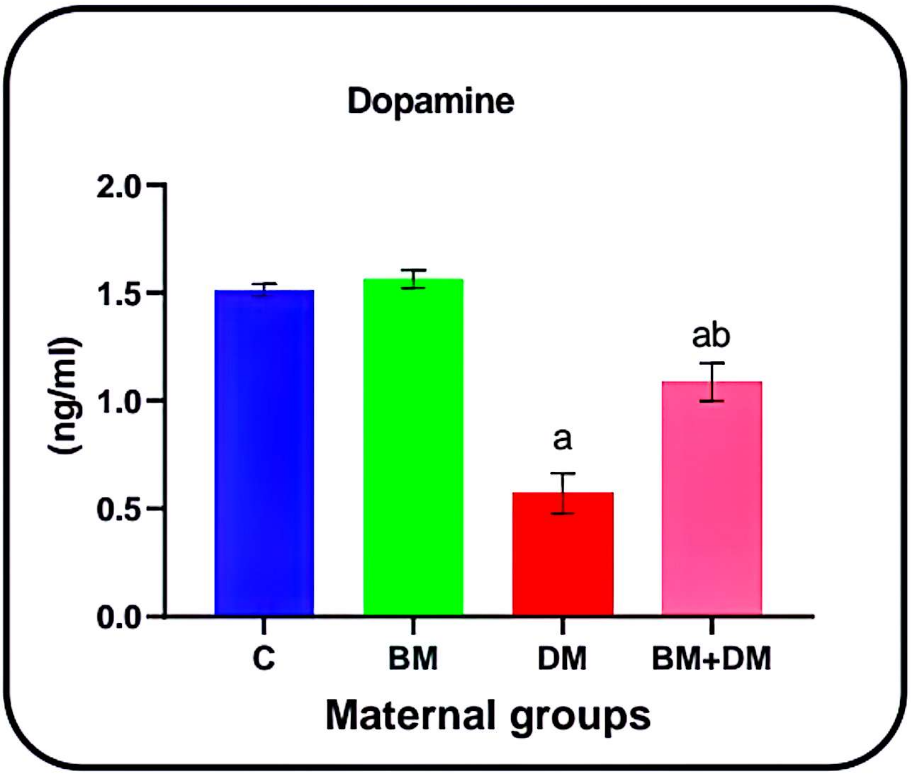

Figure (14): Serum Dopamine level (ng/ml) in control and different maternal groups.

CamScanner

Table (13): Serum Acetylcholine level (ng/ml) in control and different maternal groups.

Maternal groups		C	BM	DM	BM+DM
Mean		4.3	4.6	1.2[a]	2.3[ab]
±SE		±0.041	±0.31	±0.044	±0.035
% of change	*		6.977	-72.093	-46.512
	**				91.667

Results are presented as means ±SE and % of change.
(n=6 for each group).
a, b significant changes at $p<0.05$.
a: significant as compared to control.
b: significant as compared to DM group.
(*): % of change related to control group.
(**): % of change related to DM group.
BM: Bitter melon, DM: Diabetes Mellitus.

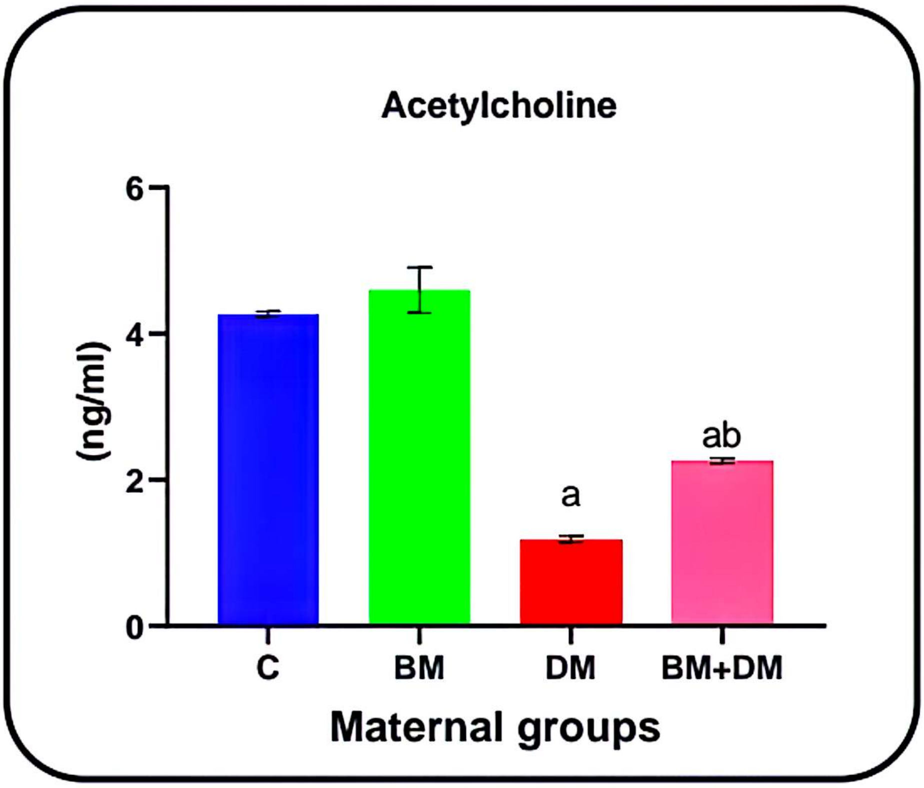

Figure (13): Serum Acetylcholine level (ng/ml) in control and different maternal groups.

CamScanner

Table (4): QUICKI in control and different maternal groups.

Maternal groups		C	BM	DM	BM+DM
Mean		0.42	0.43	0.3[a]	0.36[ab]
±SE		±0.0034	±0.011	±0.00048	±0.0039
% of change	*		2.38	-28.57	-14.29
	**				20

Results are presented as means ±SE and % of change.
(n=6 for each group).
a, b significant changes at $p<0.05$.
a: significant as compared to control.
b: significant as compared to DM group.
(*): % of change related to control group.
(**): % of change related to DM group.
BM: Bitter melon, DM: Diabetes Mellitus.

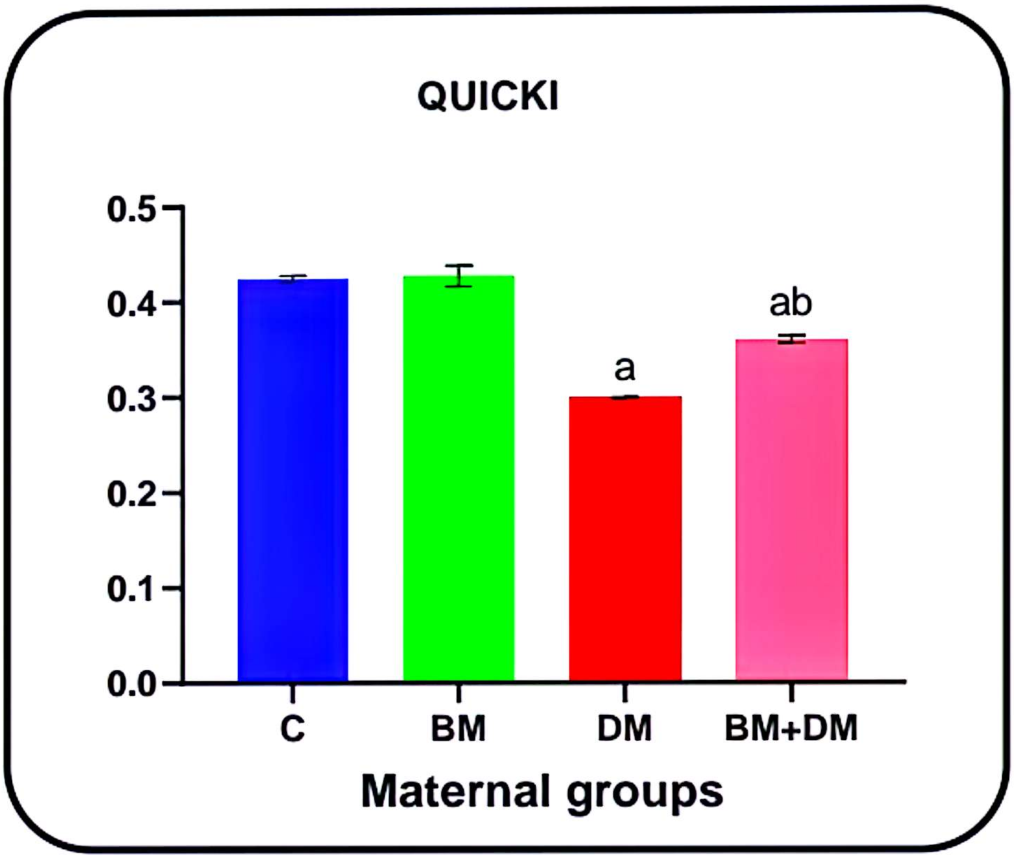

Figure (4): QUICKI in control and different maternal groups.

Table (3): HOMA IR in control and different maternal groups.

Maternal groups		C	BM	DM	BM+DM
Mean		0.56	0.55	5.5[a]	1.5[ab]
±SE		±0.024	±0.067	±0.069	±0.1
% of change	*		-1.79	882.14	167.86
	**				-72.73

Results are presented as means ±SE and % of change.
(n=6 for each group).
a, b significant changes at $p<0.05$.
a: significant as compared to control.
b: significant as compared to DM group.
(*): % of change related to control group.
(**): % of change related to DM group.
BM: Bitter melon, DM: Diabetes Mellitus.

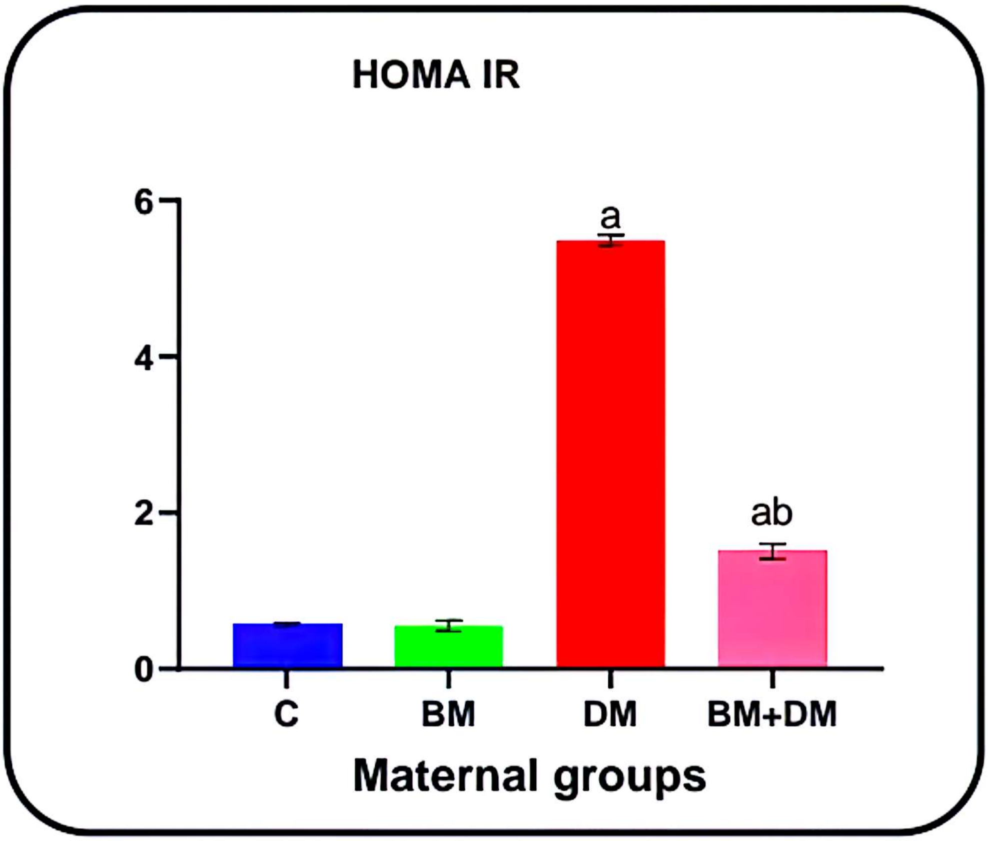

Figure (3): HOMA IR in control and different maternal groups.

CamScanner

Table (2): Serum insulin level (ng/µl) in control and different maternal groups.

Maternal groups		C	BM	DM	BM+DM
Mean		2.7	2.6	8.8[a]	4.5[ab]
±SE		±0.25	±0.22	±0.3	±0.31
% of change	*		-3.70	225.93	66.67
	**				-48.86

Results are presented as means ±SE and % of change.
(n=6 for each group).
a, b significant changes at $p<0.05$.
a: significant as compared to control.
b: significant as compared to DM group.
(*): % of change related to control group.
(**): % of change related to DM group.
BM: Bitter melon, DM: Diabetes Mellitus.

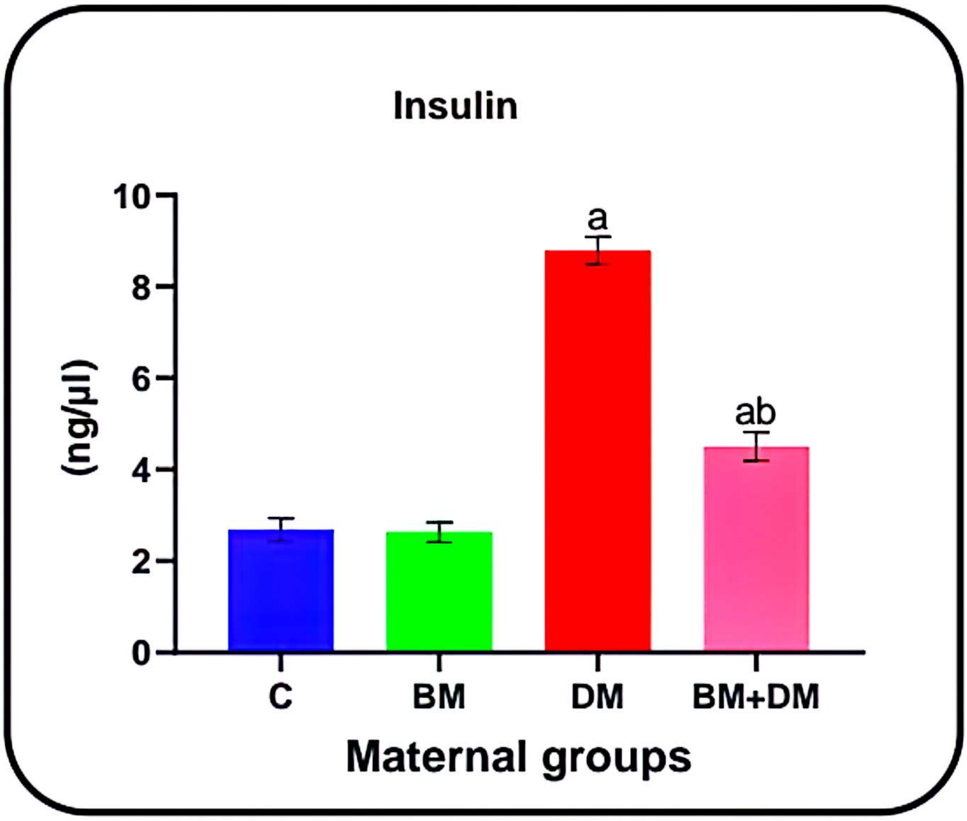

Figure (2): Serum insulin level (ng/µl) in control and different maternal groups.

CS CamScanner

RESULTS

Table (1): Serum glucose level (mg/dl) in control and different maternal groups.

Maternal groups		C	BM	DM	BM+DM
Mean		86	85	253[a]	135[ab]
±SE		±4.7	±4.4	±7.1	±3.8
% of change	*		-1.16	194.19	56.98
	**				-46.64

Results are presented as means ±SE and % of change.
(n=6 for each group).
a, b significant changes at $p<0.05$.
a: significant as compared to control.
b: significant as compared to DM group.
(*): % of change related to control group.
(**): % of change related to DM group.
BM: Bitter melon, DM: Diabetes Mellitus.

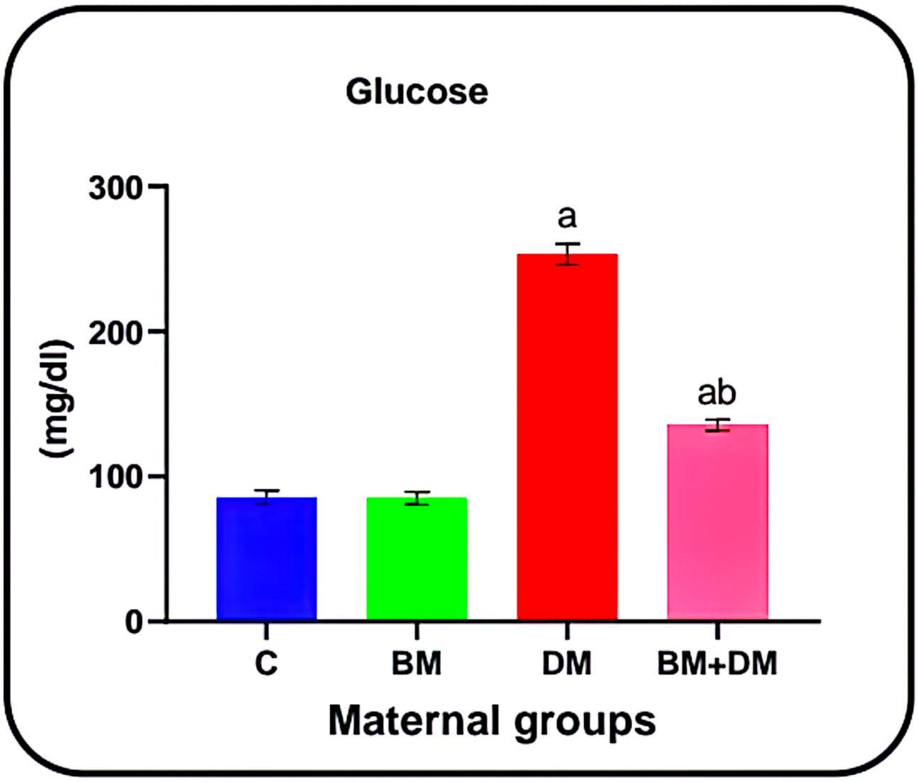

Figure (1): Serum glucose level (mg/dl) in control and different maternal groups.

CamScanner
